# K^+^ Channels in Primary Afferents and Their Role in Nerve Injury-Induced Pain

**DOI:** 10.3389/fncel.2020.566418

**Published:** 2020-09-17

**Authors:** Peter A. Smith

**Affiliations:** Department of Pharmacology and Neuroscience and Mental Health Institute, University of Alberta, Edmonton, AB, Canada

**Keywords:** neuropathic pain, nerve injury, neuroimmunology, allodynia, hyperalgesia, dorsal root ganglion, primary afferent, electrophysiology

## Abstract

Sensory abnormalities generated by nerve injury, peripheral neuropathy or disease are often expressed as neuropathic pain. This type of pain is frequently resistant to therapeutic intervention and may be intractable. Numerous studies have revealed the importance of enduring increases in primary afferent excitability and persistent spontaneous activity in the onset and maintenance of peripherally induced neuropathic pain. Some of this activity results from modulation, increased activity and /or expression of voltage-gated Na^+^ channels and hyperpolarization-activated cyclic nucleotide–gated (HCN) channels. K^+^ channels expressed in dorsal root ganglia (DRG) include delayed rectifiers (K_v_1.1, 1.2), A-channels (K_v_1.4, 3.3, 3.4, 4.1, 4.2, and 4.3), KCNQ or M-channels (K_v_7.2, 7.3, 7.4, and 7.5), ATP-sensitive channels (K_IR_6.2), Ca^2+^-activated K^+^ channels (K_Ca_1.1, 2.1, 2.2, 2.3, and 3.1), Na^+^-activated K^+^ channels (K_Ca_4.1 and 4.2) and two pore domain leak channels (K_2p_; TWIK related channels). Function of all K^+^ channel types is reduced via a multiplicity of processes leading to altered expression and/or post-translational modification. This also increases excitability of DRG cell bodies and nociceptive free nerve endings, alters axonal conduction and increases neurotransmitter release from primary afferent terminals in the spinal dorsal horn. Correlation of these cellular changes with behavioral studies provides almost indisputable evidence for K^+^ channel dysfunction in the onset and maintenance of neuropathic pain. This idea is underlined by the observation that selective impairment of just one subtype of DRG K^+^ channel can produce signs of pain *in vivo.* Whilst it is established that various mediators, including cytokines and growth factors bring about injury-induced changes in DRG function and excitability, evidence presently available points to a seminal role for interleukin 1β (IL-1β) in control of K^+^ channel function. Despite the current state of knowledge, attempts to target K^+^ channels for therapeutic pain management have met with limited success. This situation may change with the advent of personalized medicine. Identification of specific sensory abnormalities and genetic profiling of individual patients may predict therapeutic benefit of K^+^ channel activators.

## Introduction

Neuropathic pain is caused by disease or lesion of the somatosensory system (Treede et al., 2008). It can arise from a broad range of insults, including peripheral nerve trauma, post herpetic neuralgia, spinal cord injury, stroke or from neuropathies associated with chemotherapy, diabetes or HIV infection (von Hehn et al., 2012; Alles and Smith, 2018). Pain associated with fibromyalgia, osteoarthritis, complex regional pain syndromes, multiple sclerosis, rheumatoid arthritis and autoimmune disease is also dominated by neuropathic components (Olechowski et al., 2009; Lu et al., 2012; Sumpton and Moulin, 2014; Mifflin and Kerr, 2017; Rifbjerg-Madsen et al., 2017). Neuropathic pain is usually chronic and often intractable. Unlike nociceptive pain, which serves to protect an individual from potential or actual injury, neuropathic pain typically persists long after tissue healing and recovery has taken place. This and the fact that some symptoms of neuropathic pain persist in the absence of any type of sensory stimulus (Scholz and Woolf, 2007) means that this type of pain is maladaptive and serves no obvious biological purpose (Iadarola and Caudle, 1997; Costigan et al., 2009).

I will focus on neuropathic pain associated with peripheral nerve trauma and/or peripheral neuropathies as this aligns with the focus of the present collection of papers on “sensory abnormalities and primary sensory neurons.” Consideration of the K^+^ channels that play important roles in Schwann cells, satellite and other types of glial cells (Chiu and Wilson, 1989; Konishi, 1989; Olsen et al., 2015; Stebbing et al., 2015; Murata et al., 2016; Lu et al., 2017) is beyond the scope of the present review. Some of the evidence relating to the role of glial K^+^ channels in pathological pain processes can be found in the review by Tsantoulas and McMahon (2014).

### How Is Pain Measured?

In approved animal models, investigations into the etiology of neuropathic pain often involve surgical or chemical lesions to peripheral nerves followed by *in vivo* or *ex vivo* investigations of the properties of primary afferent neurons. These are correlated with behavioral studies which seek to assess pain intensity by indices such as mechanical or thermal withdrawal threshold or presence of hyperalgesia and touch-induced pain (allodynia) (Kim et al., 1997; Decosterd and Woolf, 2000; Mogil, 2009; Stemkowski and Smith, 2013). Unfortunately, these “classical” models may better reflect nociception rather than “pain” *per se* which is defined as “an unpleasant sensory and *emotional* experience associated with actual or potential tissue damage or described in terms of such damage”. It may be argued, for example, that withdrawal of a foot or limb in response to a noxious stimulus may simply reflect activation of a spinal reflex (Mogil, 2009). Thus, current models for assessment of chronic pain involve determination of indices such as facial grimace score as well as observation of social interaction and nest-building (Sotocinal et al., 2011; Turner et al., 2019). A complementary approach is to use operant models such as conditioned place preference protocols. In one version of this, rodents are required to make a conscious choice between being in a pain-inducing environment and an otherwise undesirable environment such as a brightly illuminated space (Mauderli et al., 2000; Negus et al., 2006; Alles and Smith, 2016; Harte et al., 2016). The time spent in the undesirable environment gives an index of the pain the animal is experiencing.

### Lost in Translation

In addition to the difficulty in assessing *bona fide* pain in animal models, several other considerations limit the translation between laboratory studies and beneficial human treatments. Such issues explain the slow progress in development of urgently needed new therapeutic approaches (Clark, 2016).

It is recognized, for example, that the processes initiating neuropathic pain (within 1–2 weeks of peripheral nerve injury) differ from those that maintain it over periods of months or years (Ji et al., 2006, 2019; Zhuang et al., 2006). Although the latter phenomena are more relevant to the clinical presentation of neuropathic pain, much of the work done in the laboratory has been associated with investigation of mechanisms underlying pain onset (Gold and Gebhart, 2010; Clark, 2016; Noh et al., 2020).

Two people can have virtually identical lesions and whilst one may recover, the other may develop chronic and intractable pain (Peiro et al., 2016). Genetic components must therefore play a role in human pain vulnerability (Costigan et al., 2009; Zorina-Lichtenwalter et al., 2016). This is reflected in animal studies as Fisher F-344 rats completely recover from nerve injury induced allodynia within 28 days whereas inbred Lewis and outbred Sprague-Dawley rats do not (Herradon et al., 2007). There is even considerable variation in injury-induced pain responses of different individuals of the same strain (Liu et al., 2001). Variation in pain response to injury may not only relate to hereditary genetics but also epigenetics reflecting prior exposure to noxious stimuli (Ciotu et al., 2019; Brewer and Baccei, 2020; Brewer et al., 2020; Topham et al., 2020).

Ongoing molecular genetics studies seek to identify genes that predispose certain human populations and/or individuals to pain development (Dib-Hajj and Waxman, 2014; Peiro et al., 2016). Variations in the propensity of certain individuals to develop chronic pain may even reflect differences in their gastrointestinal microbiome (Guo et al., 2019).

A further complication is that mechanisms underlying neuropathic pain in females differ from those in males (Wagner et al., 1995; Mogil, 2012; Sorge et al., 2015; Dodds et al., 2016; Mifflin et al., 2018) and women are more prone than men to develop neuropathic pain (Bannister et al., 2020). To the best of my knowledge, only one study appears to address possible differences in the effect of injury on K^+^ channel function in females versus males (Ritter et al., 2015b). This parallels the issue of species differences. It has been shown, for example that while human and rat peripheral neurons express the inwardly rectifying K^+^ channel GIRK2 (Kir3.2) these channels are not expressed in this location in mice (Nockemann et al., 2013).

Finally, the variety of peripheral nerve injury models that have been used provoke different changes in the physiology and behavioral manifestations of pain and nociception (Kim et al., 1997; Lee et al., 1998; Decosterd and Woolf, 2000; Chen et al., 2009b; Stemkowski and Smith, 2013; Noh et al., 2020). This complicates the evaluation, comparison, integration and summation of data from different research groups. It may also explain help to contradictory findings. For example, spinal nerve ligation reduces expression of mRNA for delayed rectifier K_v_1.2 channels (Fan et al., 2014) but this not seen in a model of painful diabetic neuropathy (Cao et al., 2010, see section “Effects of Nerve Injury on Delayed Rectifier K^+^ Currents and Channels in DRG Neurons”).

#### Animal Models of Neuropathic Pain

Chronic neuropathic pain and models thereof are distinct from chronic inflammatory pain which is modeled by injection of irritants such as complete Freund’s adjuvant (CFA) into the tail, paw, muscle or joint (Gregory et al., 2013). Whereas neuropathic pain models provoke axonal degeneration (Ramer et al., 1997; Ma et al., 2003), tissue inflammation associated with CFA injection may not cause damage to nerves *per se.* Although there are doubtless molectular parallels between chronic inflammatory and neuropathic pain, important diffferences have been identified (Djouhri, 2016). Space constraints limit discussion of altered K^+^ channel function in inflammation and only nerve injury effects will be considered.

The use of sciatic nerve section as a model for neuropathic pain was put forward many years ago (Wall and Gutnick, 1974) but the complete loss of sensory nerve fibers prevents testing for mechanical or thermal hyperalgesia. Whole nerve transection does however promote the phenomenon of autotomy (Wall et al., 1979). This involves attacking and gnawing the foot of the nerve transected limb. Since it has been argued that the appearance of autotomy parallels the onset of neuropathic pain in humans (Coderre et al., 1986), it may be an index of pain *per se* rather than a manifestation of altered nociception. Despite this, sciatic nerve section is only an appropriate model for post-amputation or phantom limb pain, it has largely been superseded by manipulations that better mimic more frequently observed cases of human nerve trauma.

The first of these is the chronic constriction injury model (CCI). There are several variations. The Chung model and the Bennett and Xie model which involve the positioning of ligatures around the sciatic nerve (Bennett and Xie, 1988; Kim and Chung, 1992), the Mosconi Kruger model which involves encapsulation of the sciatic nerve in a polyethylene cuff (Mosconi and Kruger, 1996) and the Seltzer model which involves ligation of half of the sciatic nerve high within the thigh (Seltzer et al., 1990).

The spared nerve injury (SNI) model was developed over 20 years ago (Decosterd and Woolf, 2000). This involves ligation and distal transection of the tibial and common peroneal nerves whilst leaving the sural nerve intact.

Lastly most studies on the effect of nerve injury on K^+^ channel function have favored the spinal nerve ligation and transection models (SNL and SNT; Table 1). These involve ligation or severing the L5 dorsal root proximal to the DRG whilst leaving the L4 root intact. Studies on the properties of the L4 DRG allow examination of the properties of uninjured neurons (Ma et al., 2003) whereas those on L5 DRG allow examination of injured neurons with severed axons.

**TABLE 1:** Known changes in K^+^ channel function in DRG following various types of peripheral nerve injury (↑, increase; ↓, decrease; ↔, unchanged; *, in large cells only; †, in medium sized cells only; §, in small IB4 positive neurons; €, in small IB4 negative neurons; $, neuron type unspecified.

*DRG K^+^ Channel Type*	*Sciatic Nerve Transection*	*Chronic Constriction Injury (CCI)*	*Spinal Nerve Transection or Ligation (SNT or SNL)*	*Other*	*Evidence for Role in Neuropathic Pain*
**Delayed Rectifiers**					
Whole cell delayed rectifier current	↓ (Everill and Kocsis, 1999; Everill and Kocsis, 2000; Abdulla and Smith, 2001b; Yang et al., 2004) *†§€			↓Diabetic Neuropathy (Cao et al., 2010)*†	K_v_ antibodies in acquired neuromyotonia patients (Hart et al., 2002)
K_v_1.1	↓mRNA (Yang et al., 2004) $	↓mRNA (Rasband et al., 2001; Kim et al., 2002) € ↓Current (Gonzalez et al., 2017) €		↔ mRNA Diabetic Neuropathy (Cao et al., 2010) *†	Expression of K_v_1.1 dominant negative causes allodynia (Hao et al., 2013). Antibodies to K_v_1 associated proteins in Morvan’s syndrome (Irani et al., 2010)
K_v_1.2	↓mRNA (Yang et al., 2004) $ ↓protein (IHC) (Ishikawa et al., 1999) € ↓protein (IHC) (Fan et al., 2014)*†	↓mRNA (Rasband et al., 2001; Kim et al., 2002) € ↓Current (Gonzalez et al., 2017) € *(see note 1)* ↓Protein (WB) (Li Z. et al., 2019)*	↓protein (IHC) (Fan et al., 2014)*† ↓mRNA (Zhao et al., 2013; Sun et al., 2019) $ ↓current (Sun et al., 2019) $	↔ mRNA Diabetic neuropathy (Cao et al., 2010) *†	Modulation of DNMT1 (Sun et al., 2019) or miR137 function (Zhang et al., 2020) altered channel function and increases or decreases in pain hypersensitivity. Knockdown of Kv1.2 by siRNA induces mechanical and thermal hypersensitivity in naive rats (Zhang et al., 2020)
K_v_1.3	↓mRNA (Yang et al., 2004) $				
K_v_1.5	↔ mRNA (Yang et al., 2004) $	↔ mRNA (Kim et al., 2002) $			
K_v_1.6	↔ mRNA (Yang et al., 2004) $	↔ mRNA (Kim et al., 2002) $			
K_v_2.1	↓protein (IHC) (Ishikawa et al., 1999) €	↔ mRNA (Kim et al., 2002) $	↓mRNA(Tsantoulas et al., 2014) € ↓current (Tsantoulas et al., 2014) *	↔ mRNA Diabetic Neuropathy (Cao et al., 2010) *†	
K_v_2.2		↓mRNA (Kim et al., 2002) $	↓mRNA(Tsantoulas et al., 2014) € ↓current (Tsantoulas et al., 2014) *	↔ mRNA Diabetic Neuropathy (Cao et al., 2010) *†	
K_v_3.1		↔ mRNA (Kim et al., 2002) $			
K_v_3.2		↔ mRNA (Kim et al., 2002) $			
K_v_6.1 K_v_8.1	*No data available*				
K_v_9.1			↓Protein and mRNA by IHC and ISH (Tsantoulas et al., 2012)*		siRNA-mediated knock-down of K_v_9.1 in naive rats leads to neuropathic pain behaviors (Tsantoulas et al., 2012)
Kv9.2	No data available				
Kv9.3					
**KCNQ/Kv7.2/7.3/M-channels**					
Whole cell M-current		↓(Rose et al., 2011) §€		↓Bone cancer pain (Zheng et al., 2013) §€	
Kv7.2		↓mRNA and protein WB and IHC (Rose et al., 2011) §€		↓Protein (IHC) Bone cancer pain (Zheng et al., 2013) §€	Kv7.2 knockout in DRG causes hyperalgesia (King et al., 2014) G9a inhibitors attenuate pain hypersensitivity (Laumet et al., 2015) Gain of function mutation in Kv7.2 reduces pain in “burning man syndrome” (Mis et al., 2019)
Kv7.3				↓Protein Bone cancer pain (Zheng et al., 2013) §€	Anti-allodynic effect of retigabine (Blackburn-Munro and Jensen, 2003; Dermody et al., 2012; Djouhri et al., 2019)
**A-currents**					
Whole cell A-current	↓ (Yang et al., 2004) §€ ↓ (Everill and Kocsis, 1999; Everill and Kocsis, 2000)*			↑Bone cancer pain (Duan et al., 2012) § ↓Diabetic neuropathy (Cao et al., 2010; Grabauskas et al., 2011) *†	miR-17-92 overexpression downregulates A-channels and promotes hyperalgesia (Sakai et al., 2017). G9a inhibitors attenuate pain hypersensitivity (Laumet et al., 2015)
Kv1.4 (N-type inactivating)		↓mRNA (Kim et al., 2002) €	↔ mRNA (Sun et al., 2019) $	↓mRNA Diabetic Neuropathy (Cao et al., 2010) *† ↑Protein (IHC) Bone cancer pain (Duan et al., 2012) §	siRNA knockdown of Kv1.4 causes allodynia (Duan et al., 2012)
Kv3.3	No data available				
Kv3.4 (N-type inactivating and highly sensitive to 4-AP)			↓Immunoreactivity (Chien et al., 2007) §	↓mRNA Diabetic Neuropathy (Cao et al., 2010) *† ↑Protein (IHC) Bone cancer pain (Duan et al., 2012) §	Kv3.4 antisense produces mechanical hypersensitivity (Chien et al., 2007)
Kv4.1		↔ mRNA (Kim et al., 2002) $			Knockdown of Kv4.1 and modulatory subunits causes mechanical hypersensitivity (Kuo et al., 2017)
Kv4.2		↓mRNA (Kim et al., 2002) $		↓mRNA Diabetic Neuropathy (Cao et al., 2010) *† Decreased function by phosphorylation (Grabauskas et al., 2011)	
Kv4.3		↓mRNA (Kim et al., 2002) $ ↓Protein expression WB (Kuo et al., 2017) §	↓Immunoreactivity (Chien et al., 2007) §	↓mRNA Diabetic Neuropathy (Cao et al., 2010). *† ↑Protein Bone cancer pain (Duan et al., 2012) §	
**Calcium (and sodium) activated K^+^ channels**					
Whole cell apamin sensitive (SK) current			↓↑ (Sarantopoulos et al., 2007) (see note 2) †		
KCa2.1 (apamin sensitive SKCa1)		↔Protein by WB and IHC (Mongan et al., 2005) §€		↓protein (IHC) in DRG of pain patients (Boettger et al., 2002) $	
KCa2.2 (apamin sensitive SKCa2)		↔Protein by WB and IHC (Mongan et al., 2005) §€			
KCa2.3 (apamin sensitive SKCa3)	No data available				
KCa3.1 (apamin insensitive, clotrimazole sensitive intermediate conductance IAHP or IKCa1)		↔Protein by WB and IHC (Mongan et al., 2005) §€		↓protein (IHC) in DRG of pain patients (Boettger et al., 2002) $	
Whole cell BK current (Ic)	↓ as a result of decreased Ca2^+^ influx (Abdulla and Smith, 2001b)		↓↑ (Sarantopoulos et al., 2007) (see note 1)\ ↓(Cao et al., 2012)†*		G9a inhibitors attenuate pain hypersensitivity (Laumet et al., 2015).
KCa1.1 (Slo1, BK or Maxi gK, Ca)			↓KCNMA1 expression (Laumet et al., 2015). $ ↓mRNA and protein by WB and IHC (Chen et al., 2009a) †§€		Iberiotoxin reduction of mechanical threshold and relief of SNL induced allodynia with BK activator (Chen et al., 2009a)
KCa4.1 (Na^+^ dependent K^+^ channel; Slo 2.2 or slack)	No data available				Loxapine activates KCa4.1 and alleviates signs of neuropathic pain in SNI model (Lu et al., 2015)
KCa4.2 (Na^+^ dependent K^+^ channel; Slo 2.1 or slick)	No data available				
KCa5.1 (pH sensitive K^+^ channel)	No data available				
**Two transmembrane-domain, inwardly rectifying K^+^ channels (Kir)**					
Kir2.1,	No data available				
Kir2.2					
Kir2.3					
Kir3.1 (GIRK1) Kir3.2 (GIRK2)	↓mRNA and protein by IHC and WB (Lyu et al., 2015) $				
Kir 3.3 (GIRK 3)	No data available				
Kir 3.4 (GIRK 4)					
KATP Channel Activity			↓(Kawano et al., 2009)*		
Kir6.1	No data available				Anti-nociceptive actions of KATP channel openers (Vergoni et al., 1992; Welch and Dunlow, 1993)
Kir6.2					
**Two Pore domain K^+^ channels**					
K2p1 (TWICK1)				↓mRNA and protein (IHC) (Pollema-Mays et al., 2013) (Spared Nerve Injury; SNI) * †	
K2p3 (TASK1)				↔ mRNA and protein (IHC) (Pollema-Mays et al., 2013) (Spared Nerve Injury; SNI) * †	
K2p9 (TASK2)				↓mRNA and protein (IHC) (Pollema-Mays et al., 2013) (Spared Nerve Injury;SNI) * †	
K2p18 TRESK channels	↓mRNA (Tulleuda et al., 2011)				*In vivo* knock down decreases threshold to painful mechanical stimuli (Tulleuda et al., 2011; Castellanos et al., 2020)

*The last column summarizes evidence for involvement of the various DRG K^+^ channel types in neuropathic pain. Notes: (1) In TRPM8 expressing neurons. (2) Sarantopoulos et al. (2007) showed in an SNL model that BK conductance decreased in medium sized neurons where axons were actually severed (axotomized L5 DRG) whereas BK channel function was increased in adjacent undamaged neurons (L4 DRG). By contrast, in small neurons BK current was reduced in both axotomized (L5) and adjacent (L4) neurons.*

In the CCI, SNL, SNT, and SNI models some axons are subject to Wallerian degeneration whereas others remained relatively unscathed (Ramer et al., 1997; Ma et al., 2003). The release of inflammatory mediators and growth factors from the site of injury is thought to initiate changes in axons and cell bodies of both injured and uninjured axons (Wagner and Myers, 1996a,b; Sorkin et al., 1997; Sommer et al., 1999; Cui et al., 2000; George et al., 2004). Because a fraction of both sensory and motor axons survive after SNL, SNT, CCI and SNI, animals can be assessed for mechanical and/or thermal hyperalgesia. The main difference between CCI and SNI is that the behavioral responses to CCI subside within a month or so whereas those produced by SNI persist for much longer periods of time (Decosterd and Woolf, 2000; Noh et al., 2020). SNL, SNT, and SNI are thus emerging as preferred animal protocols by which to model human neuropathic pain.

## Role of Increased Peripheral Neuron Excitability and Dorsal Root Ganglia in Neuropathic Pain

Since the concept was first put forward (Govrin-Lippmann and Devor, 1978; Wall and Devor, 1983), an overwhelming body of evidence now supports the indispensable role of peripheral nerve sensitization and ongoing, aberrant spontaneous activity in the onset and persistence of pain following neuropathy or peripheral nerve injury (Costigan et al., 2009; Bedi et al., 2010; Gold and Gebhart, 2010; Sexton et al., 2017; Koplovitch and Devor, 2018; Waxman, 2019; Yatziv and Devor, 2019; Yousuf et al., 2019; Waxman, 2019). Much of this peripheral sensitization results from the dorsal root ganglia (DRG) *per se* as well as from nerve axons or terminals and/or the site of injury (Kajander et al., 1992; Yatziv and Devor, 2019). Aberrant spontaneous activity in injured peripheral nerves is thought to drive the sensitization of spinal circuits; an established harbinger of chronic pain (Woolf, 1983; Sandkuhler, 2009; Gold and Gebhart, 2010; Latremoliere and Woolf, 2009; Tsantoulas and McMahon, 2014; Alles and Smith, 2018).

Intracellular recordings from DRG neurons both *ex vivo* and *in vitro* confirmed that peripheral nerve injury increases their excitability and may provoke spontaneous discharge of action potentials (Study and Kral, 1996; Djouhri et al., 2018). In a study of the effect of complete nerve section, we found and that the most profound increases were seen in animals that displayed autotomy (Abdulla and Smith, 2001a). If it is accepted that the appearance of autotomy parallels the onset of neuropathic pain (Coderre et al., 1986), our findings support a role for increased DRG excitability in the etiology of pain *per se* as opposed to a simple consequence of altered nociception. In a complementary study, it was found that individual rats with a high propensity to exhibit autotomy following peripheral nerve injury exhibited more spontaneous primary afferent activity than those with a weaker autotomy phenotype (Liu et al., 2001).

We also found that peripheral nerve chronic constriction injury (CCI) increases the excitatory synaptic drive to putative excitatory *substantia gelatinosa* neurons (Balasubramanyan et al., 2006; Lu et al., 2009). Although intrinsic properties of *substantia gelatinosa* neurons such as excitability, rheobase and input resistance were unchanged, the frequency and amplitude of spontaneous excitatory synaptic events was increased. As will be discussed below, this may be a consequence of altered expression of K^+^ channels in primary afferent nerve terminals (Barkai et al., 2017; Muqeem et al., 2018). This is again consistent with the notion that injury induced-sensitization of spinal circuits is driven by, and attributed to changes in primary afferent activity (Devor, 2006). This idea is supported further by the observation that peripheral nerve injury increases the spontaneous discharge rate of wide dynamic range (WDR) neurons in the rat dorsal horn and that this effect is attenuated by peripheral application of lidocaine (Pitcher and Henry, 2008).

More recent work continues to underline the role of aberrant and persistent peripheral nerve activity in the long-term sensitization of spinal nociceptive circuits following nerve injury. Thus, application of low dose lidocaine to DRG within the intervertebral foramen transiently suppresses allodynia in a rat SNL model. Normal nerve conduction was unaffected and sustained infusion of low dose lidocaine for 2 weeks using osmotic minipumps suppressed allodynia for the duration of the infusion. Since application of low dose lidocaine to the peripheral site of injury to the sciatic nerve was ineffective, these findings not only support a role for spontaneous peripheral nerve activity in pain generation but suggest that such activity originates in the DRG (Yatziv and Devor, 2019). This study was based on the previous electrophysiological observation that low concentrations of lidocaine suppress ectopic neuropathic discharge in dorsal root ganglia without blocking propagation of action potentials along the axon (Koplovitch and Devor, 2018).

Another study used *in vivo* optogenetic methodology to silence nociceptive primary afferents which express the voltage gated Na^+^ channel Na_v_1.8. This was achieved by selectively expressing inhibitory archaerhodopsin-3 (Arch) proton pumps in Na_v_1.8(+) neurons. Activation of Arch by yellow light leads to membrane hyperpolarization. Acute transdermal illumination of the hindpaw of Na_v_1.8(+)-Arch(+) mice *in vivo* reduced mechanical allodynia under inflammatory conditions, while basal mechanical sensitivity was unaffected. Furthermore, in mice made neuropathic by SNI, prolonged optical silencing of Na_v_1.8(+) peripheral afferents led to lasting analgesia with a decrease in mechanical and thermal hypersensitivity (Daou et al., 2016).

Confirmation of the role of persistent increased excitability of peripheral neurons in a clinical setting was obtained from examination of amputees who suffered phantom limb pain (Vaso et al., 2014). Application of lidocaine intrathecally or to the DRG surface during intraforaminal epidural block reversibly extinguished phantom limb pain. Similar results in amputees have been reported using peripheral nerve block (Buch et al., 2019). Indeed, lidocaine patches still retain a role, albeit a minor one, in the management of neuropathic pain in adults (Finnerup et al., 2015).

### Which Types of Sensory Neuron Are Affected?

Classically, Aβ fibers convey tactile and non-noxious sensation, Aδ fibers convey high threshold mechanical and thermal sensation (first pain) and polymodal C-fibers convey thermal mechanical and inflammatory pain (second pain). More recent studies have illustrated that the situation is much more complex (Snider and McMahon, 1998; Lawson, 2002; Gold and Gebhart, 2010; Chiu et al., 2014; Peirs and Seal, 2016; Arcourt et al., 2017; Zheng et al., 2019; Giacobassi et al., 2020). C-fibers are known to fall into at least two categories; nociceptors that contain neuropeptides such as substance P and calcitonin gene related peptide (CGRP) and neurons which bind the plant lectin IB4 and often express Ret, the receptor for the glial cell line-derived family of neurotrophins as well as the purinergic P2X3 receptor (Stucky and Lewin, 1999; Fang et al., 2006; Gold and Gebhart, 2010). It is suggested that IB4-positive nociceptors play a role in mediating mechanical inflammatory hypersensitivity rather than normal mechanical nociception (Pinto et al., 2019).

Although DRG neurons are heterogeneous and highly specialized (Zheng et al., 2019; Giacobassi et al., 2020) the simple answer to the question of which neuronal types are affected by nerve injury is “probably all of them depending on the nature of the injury” (see Table 2).

**TABLE 2:** Effects of injury and inflammatory mediators on K+ channel function in Large, Medium, Small IB4 positive and Small IB4 negative neurons.

Treatment	Large Neurons	Medium Neurons	Small IB4 Positive Neurons	Small IB4-negative Neurons
Sciatic Nerve Transection	↑ Spike Width and excitability (Abdulla and Smith, 2001a) ↓ Delayed Rectifier and gK, Ca (Abdulla and Smith, 2001b) ↓ Transient and persistent K+ current (Everill and Kocsis, 1999; Everill and Kocsis, 2000)	↑ Spike Width and excitability (Abdulla and Smith, 2001a) ↓ Delayed Rectifier and gK, Ca (Abdulla and Smith, 2001b)	↑ Spike Width and excitability (Abdulla and Smith, 2001a) ↓ Whole cell A-current (Yang et al., 2004) ↓ Delayed Rectifier and gK, Ca (Abdulla and Smith, 2001b)	
	Kv1.2, Kv2.1 Kv2.1 Kv 2.2 ↓protein (IHC) (Fan et al., 2014).	Kv1.2↓protein (IHC) (Fan et al., 2014)		Kv1.2 ↓protein (IHC) (Ishikawa et al., 1999)
CCI (Chronic Constriction Injury)	Kv 1.2 ↓Protein (WB) (Li Z. et al., 2019)			Kv1.1 ↓mRNA (Rasband et al., 2001; Kim et al., 2002) € Kv1.1 ↓Current (Gonzalez et al., 2017) € Kv1.2 ↓mRNA (Rasband et al., 2001; Kim et al., 2002) Kv1.2 ↓Current (Gonzalez et al., 2017) Kv1.4 mRNA↓ (Kim et al., 2002) Kv4.3 ↓Protein expression WB (Kuo et al., 2017)
			↓ Whole Cell M-current. Kv7.2 mRNA and protein (Rose et al., 2011), KCa2.1 KCa2.2 KCa 3.1 Protein ↔ by WB and IHC (Mongan et al., 2005)	
SNI (Spared Nerve Injury)	K2p1, K2p3. K2p9 ↓mRNA and protein (IHC) (Pollema-Mays et al., 2013)	K2p1, K2p3. K2p9 ↓mRNA and protein (IHC) (Pollema-Mays et al., 2013)		
SNT or SNL (Spinal Nerve Transection or Ligation)	Kv1.2 ↓protein (IHC) (Fan et al., 2014) Kv2.1, Kv 2.2 ↓current (Tsantoulas et al., 2014) ↓Protein and mRNA by IHC and ISH (Tsantoulas et al., 2012)* ↓Whole cell BK current (Cao et al., 2012) ↓ K_ATP_ channel activity (Kawano et al., 2009)	Kv1.2 ↓protein (IHC) (Fan et al., 2014) Whole cell apamin sensitive and BK current ↓↑ (Sarantopoulos et al., 2007) *(see note 2 in Table 1)* ↓ Whole cell BK current (Cao et al., 2012) ↓KCa1.1 mRNA and protein by WB and IHC	↓KCa1.1 mRNA and protein by WB and IHC	Kv 2.2 ↓mRNA (Tsantoulas et al., 2014) Kv3.4, Kv4.3 ↓Immunoreactivity (Chien et al., 2007) ↓KCa1.1 mRNA and protein by WB and IHC
Other	Kv1.4 Kv 3.4, Kv4.2, Kv 4.3 mRNA↓; Kv1.1, Kv1.2, Kv2.1 Kv 2.2 ↔ mRNA Diabetic Neuropathy (Cao et al., 2010), Whole cell A-current ↓Diabetic neuropathy (Cao et al., 2010; Grabauskas et al., 2011)	Kv1.4 Kv 3.4, Kv4.2, Kv 4.3 mRNA↓; Kv1.1, Kv1.2, Kv 2.1, Kv 2.2 ↔ mRNA Diabetic Neuropathy (Cao et al., 2010) Whole cell A-current ↓Diabetic neuropathy (Cao et al., 2010; Grabauskas et al., 2011)	Whole cell A-current and Kv1.4 Kv 3.4 protein ↑Bone cancer pain (Duan et al., 2012)	
			M-current Kv 7.2 and Kv 7.3 ↓Protein (IHC) Bone cancer pain (Zheng et al., 2013)	
IL-1β	Decreased Excitability (5–6 days IL-1β exposure) (Stemkowski and Smith, 2012a)	Increased Excitability, reduced delayed rectifier, BK and A-currents (5–6 days IL-1β exposure) (Stemkowski and Smith, 2012a; Stemkowski et al., 2015)	Increased Excitability, decreased BK current, increased TTX sensitive Na+ current (5–6 days IL-1β exposure) (Stemkowski and Smith, 2012a; Noh et al., 2019)	No effect on excitability (5–6 days IL-1β exposure) (Stemkowski and Smith, 2012a)
			Increased excitability and increased sodium channel function (acute application) (Binshtok et al., 2008)	
TNF α			Increased excitability and increased TTX resistant sodium channel function (acute application) (Jin and Gereau, 2006; Gudes et al., 2015)	
NGF	NGF restores transient and persistent K^+^ currents attenuated by sciatic nerve section (Everill and Kocsis, 2000)			Reduced total K+ current via p75 dependent process (Zhang Y. H. et al., 2012)
BDNF		Decreased BK current (Cao et al., 2012)	Decreased BK current (Cao et al., 2012)	
		Reduced expression of Kv42 and Kv4.3 in whole DRG (Park et al., 2003) BDNF antibodies or blockers increase Kv4.2n and 4.3 mRNA and A-type current		

*For studies where small neurons were not sub categorized, findings on IB4 positive and IB4 negative neurons are pooled.*

By contrast with nociceptive pain which is primarily transmitted by Aδ and C-fibers, there is good evidence to support a role of Aβ afferents in neuropathic pain in both animal models (Devor, 2009; Sandkuhler, 2009; Tashima et al., 2018) and in clinical situations (Campbell et al., 1988). One aspect of central sensitization is loss of inhibition (Coull et al., 2003) which enables transfer of aberrant excitation between deeper spinal cord laminae and more superficial laminae (Baba et al., 2003; Schoffnegger et al., 2008; Peirs et al., 2015; Peirs and Seal, 2016; Boyle et al., 2019). Because the deeper laminae are primarily involved in the processing of tactile information and superficial laminae process nociceptive information, it is easy to see how activation of tactile Aβ fibers can lead to allodynia as tactile information is misdirected to the superficial dorsal horn. In view of this, the following discussion of effects of injury on K^+^ channels will include those in all DRG cell types as perturbation of any of them could be potentially involved in neuropathic pain. In addition to this, there is now evidence that some Aβ fibers play a role in “normal” pain perception (Arcourt et al., 2017) and that they may undergo a “phenotypic switch” following injury wherein they acquire the properties and sensitivities of afferent C-fibers (Abdulla et al., 2001; Nitzan-Luques et al., 2011).

### Spinal Cord Injury and Changes in Peripheral Neuron Excitability

There is now evidence that pain following injury to the spinal cord *per se* (as opposed to CCI, sciatic nerve transection or SNI of peripheral nerves as discussed above) also depends upon continuing hyperactivity of *peripheral* sensory neurons (Bedi et al., 2010; Yang et al., 2014). Thus, spinal contusion injury promotes persistent upregulation of protein for the voltage-gated Na^+^ channel, Na_v_1.8. Since this channel is expressed almost exclusively in primary afferent neurons, Yang et al. (2014) were able to reverse hypersensitivity of hindlimb withdrawal reflexes, and reduced ongoing pain by knocking down Na_v_1.8 after spinal injury. Pain (as opposed to nociception) assessed was by a conditioned place preference test.

With regard to the present focus on K^+^ channels, cervical unilateral spinal cord injury has also shown to reduce surface expression of K_v_3.4 channels (A-type) in DRG neurons (Ritter et al., 2015a). It is likely therefore that inflammatory mediators produced as a result of spinal cord injury diffuse and affect channel expression and function in the DRG [see section “Distribution of High Threshold, N-type Inactivating, 4-AP and TEA Sensitive A-currents (K_v_3.4) and Effects of Nerve Injury” below].

## K^+^ Channels in Primary Afferent Neurons; Distribution and Effects of Injury

As summarized in Table 1, the majority of studies show that the function of all types of K^+^ channels is reduced following peripheral nerve injury (Everill and Kocsis, 1999; Everill and Kocsis, 2000; Abdulla and Smith, 2001b; Rasband et al., 2001; Kim et al., 2002; Park et al., 2003; Ocana et al., 2004; Kawano et al., 2009; Cao et al., 2010; Takeda et al., 2011; Tsantoulas and McMahon, 2014; Zemel et al., 2018). Many of the early studies describe attenuation of voltage-gated K^+^ conductances in the DRG cell body *per se*. In most cases, this does not lead to depolarization but may rather affect the accommodation of action potential discharge in response to persistent depolarization (Abdulla and Smith, 2001a). Spike width (action potential duration) is also increased (Gurtu and Smith, 1988; Stebbing et al., 1999; Abdulla and Smith, 2001a) and this may involve attenuation of voltage-gated K^+^ conductances. If this situation also applies to channels in primary afferent terminals, this may lead to increased glutamate release in the dorsal horn of the spinal cord (Muqeem et al., 2018).

It should be pointed out, however, that extrapolation of findings in DRG cell bodies to K^+^ channel function and distribution throughout the axons, nerve terminals and free nerve endings of the whole neuron is very much an oversimplification of processes underlying changes in excitability. The review by Tsantoulas and McMahon (2014) presents detailed information regarding K^+^ channel location, particularly in the nodal, paranodal and juxtaparanodal regions of axons. The idea that specific types of K^+^ channels are trafficked to specific subcellular regions is borne out by the observation that injury-induced early loss of K_v_1.1 and 1.2 (delayed rectifier) channels at the juxtaparanodal regions of sensory axons may be later followed by appearance of K_v_1.4 (A-type) and K_v_1.6 channels (Calvo et al., 2016). On the other hand, it should be reiterated that the DRG themselves play an important role in generating ectopic activity after nerve injury (Kajander et al., 1992; Yatziv and Devor, 2019).

Experiments using the skin-nerve preparation that allows single fiber recording from sensory afferents of excised rat skin (Reeh, 1988; Schutze et al., 2016) have shown that sensory nerve endings are excited by the K^+^ channel blockers, TEA and 4AP (Kirchhoff et al., 1992). Selective blockade of nerve ending K^+^ channels increased spontaneous activity in Aβ, Aδ and C-fibers. Other studies have identified TREK-1 (K_2p_2) in sensory C-fibers of both IB4-positive and IB4-negative neurons where it appears as an important ion channel for regulation of polymodal pain (Alloui et al., 2006). TRAAK (K_2p_4) channels have also been detected in C-fibers (Noel et al., 2009) and K_v_7.2 and 7.3 channels are expressed in the endings of D-hair Aδ fiber mechanoceptors (Schutze et al., 2016). While is it not yet known whether injury alters K^+^ channel expression in nerve endings in a similar fashion to cell bodies, a role for these channels in neuropathic pain is implied by three observations; (1) in some, but not all cases, nerve injury downregulates both mRNA (Rasband et al., 2001; Kim et al., 2002; Yang et al., 2004) and/or channel protein (Ishikawa et al., 1999; Fan et al., 2014). In these cases, K^+^ channel function would be altered at all loci within the neuron (including free nerve endings); (2) K^+^ channel function is impaired by various inflammatory mediators (Binshtok et al., 2008; Takeda et al., 2008; Stemkowski and Smith, 2012a; Stemkowski et al., 2015) most of which are released from damaged axons and Schwann cells in the vicinity of nerve endings (Sommer and Kress, 2004; Scholz and Woolf, 2007); (3) many types of K_2p_ channels are mechanosensitive (Goldstein et al., 2005) and may therefore play a role in the development of nerve injury-induced allodynia.

Whereas studies on voltage-gated Na^+^ channels have paved the way for the development of new therapeutic targets (Dib-Hajj et al., 2010; Waxman and Zamponi, 2014; Dib-Hajj and Waxman, 2019), studies on K^+^ channels have been less encouraging. Voltage-gated Na^+^ channels are monomeric proteins resulting from the nine gene products Na_v_1.1 to Na_v_1.9 (Goldin et al., 2000). Since Na_v_1.7, 1.8, and 1.9 are highly expressed in DRG and have been implicated in neuropathic pain, they present attractive targets for drug development. Because these channels are much less abundant in other excitable tissues, this limits the side effect profile of drugs which target them (Dib-Hajj et al., 2013; Zakrzewska et al., 2017). Na_v_1.3 represents another attractive target as it only appears in adult DRG neurons after nerve injury (Samad et al., 2013; Waxman and Zamponi, 2014).

Hyperpolarization activated cyclic nucleotide gated cation (HCN) channels also display increased activity following nerve injury (Chaplan et al., 2003; Smith et al., 2015; Bernal and Roza, 2018; Djouhri et al., 2018) and may be involved in pain associated with diabetic neuropathy (Tsantoulas et al., 2017) are under consideration as therapeutic targets. For example, clinically approved drugs such as the antianginal agent ivabradine are effective in attenuating allodynia in animal models (Emery et al., 2011, 2012; Noh et al., 2014; Young et al., 2014; Tsantoulas et al., 2016). HCN2 channels are a particularly attractive target as this isoform is found predominantly in neurons (Tsantoulas et al., 2016).

By contrast, many of the K^+^ channel types found in primary afferent neurons are found in many tissues and cell types throughout the body in both excitable and non-excitable cells. This may limit the therapeutic potential of K^+^ channel activators.

The 78 K^+^ channel genes so far identified in the human genome give rise to four broad families of channels (Ocana et al., 2004; Goldstein et al., 2005; Gutman et al., 2005; Kubo et al., 2005; Wei et al., 2005).

(1) the six transmembrane-domain voltage gated (K_v_) channels which assemble as homo- or hetero-tetramers from 40 or so human genes. These give rise to A-currents and delayed rectifier conductances;
(2) Ca^2+^ activated K^+^ (K_Ca_) channels which often contribute to action potential duration and afterhyperpolarization. This category includes Na^+^ sensitive K^+^ channels such as “slick” (sequence like an intermediate calcium channel) and “slack” (Wei et al., 2005);
(3) two transmembrane-domain inwardly rectifying K^+^ channels (K_IR_) which also assemble as tetramers and include instantaneous inward rectifiers as well as ATP sensitive and G-protein activated channels (Kubo et al., 2005);
(4) four transmembrane-domain tandem pore domain (K_2p_) channels which assemble as dimers and account for K^+^ leak conductance (Goldstein et al., 2005; Enyedi and Czirjak, 2010).

Representatives of all four K^+^ channel families are found in DRG neurons (McFarlane and Cooper, 1991; Gold et al., 1996a; Abdulla and Smith, 2001b; Talley et al., 2001; Kang and Kim, 2006; Kawano et al., 2009; Zemel et al., 2018; Gada and Plant, 2019) and the distribution of genetically defined channel phenotypes in various DRG neuron types is an active area of investigation (Chien et al., 2007; Bocksteins et al., 2009, 2012; Tsantoulas et al., 2014; Ritter et al., 2015b; Zemel et al., 2018). Some of these studies also address how the distribution and function of these channels is altered by nerve injury. Other work examines the molecular mechanism of altered K^+^ channel function and how alteration of function relates to pain (Chien et al., 2007; Takeda et al., 2008; Cao et al., 2010; Rose et al., 2011; Stemkowski et al., 2015; Calvo et al., 2016; Zemel et al., 2017, 2018; Djouhri et al., 2019; Noh et al., 2019). The hope is that a single K^+^ channel type or subtype will emerge as a therapeutic target in neuropathic pain in a comparable fashion to Na_v_1.7. To explore this possibility, the following sections outline the current state of knowledge regarding the location and subunit composition of various types of K^+^ channels in sensory neurons and how they affected by nerve injury. Although a considerable amount of information is available, it should be remembered that different authors have used different criteria for definition of neuronal types or have gathered information from unidentified DRG neurons in culture.

### Distribution of Delayed Rectifier K^+^ Channels in Primary Afferent Neurons

Membrane conductances mediated by delayed rectifier channels activate in response to depolarization and show little or no inactivation. They comprise hetero- or homo-tetramers from the gene products K_v_1.1, 1.2, 1.3, 1.5, 1.6, 1.7, 1.8, 2.1, 2.2, 3.1, 3.2, 7.1, 7.2, 7.3, 7.4, 7.5, 10.1, 10.2, 11.2, 11.3, 12.1, 12.2, 12.3 from the *KCNA, KCNB, KCNC, KCNQ*, and *KCNH* gene families (Gutman et al., 2005).

K^+^ channel conductances that display delayed outward rectification have been described in all types of DRG neuron (Akins and McCleskey, 1993; Gold et al., 1996a; Everill et al., 1998; Abdulla and Smith, 2001b). mRNA for K_v_1.1, K_v_1.2, K_v_1.3, K_v_1.5, and K_v_1.6 has been detected in rat DRG (Yang et al., 2004) and K_v_1.2, K_v_2.1, and K_v_2.2 mRNA appears to particularly abundant in medium and/or large sized DRG neurons (Fan et al., 2014; Tsantoulas et al., 2014). Other work has demonstrated the presence of K_v_3.1 protein and K_v_3.1 currents in small DRG neurons but these only contribute to about 19% of the delayed rectifier current (Bocksteins et al., 2012). This is consistent with the suggestion that K_v_3 channels are mainly found in the CNS (Weiser et al., 1994). The silent subunit K_v_9.1 (*KCNS1*) is present in large DRG neurons with myelinated axons but is absent from small neurons (Tsantoulas et al., 2012, 2018).

#### Effects of Nerve Injury on Delayed Rectifier K^+^ Currents and Channels in DRG Neurons

The known effects of peripheral nerve injury on all types of K^+^ channels in DRG neurons are summarized in Tables 1, 2. Delayed rectifier currents in cell bodies of small, medium and large sized DRG neurons are reduced by about 60% following sciatic nerve section (Abdulla and Smith, 2001b). mRNA’s for K_v_1.1, 1.2, and 2.2 but not K_v_1.5 and 1.6 are downregulated (Rasband et al., 2001; Kim et al., 2002; Yang et al., 2004) and immunohistochemical (IHC) studies confirm the loss of K_v_1.2 protein (Ishikawa et al., 1999; Fan et al., 2014). More recent studies have confirmed that spinal nerve transection (SNT) downregulates K_v_2.2 as well as K_v_2.1 mRNA and this may have accounted for a shortening of action potential afterhyperpolarization and increased excitability of myelinated medium to large cells (Tsantoulas et al., 2014).

Knockdown of K_v_1.2 by siRNA induces significant mechanical and thermal hypersensitivity in naive rats (Zhang et al., 2020) and considerable progress has been made in understanding the underlying epigenetic mechanisms. Three of these involve alterations in DNA methylation.

Firstly, SNL increases expression of the canonical maintenance methyltransferase DNMT1 via a CREB (cAMP response element binding protein) – dependent process. This leads to downregulation of the *KCNA2* gene, reduced *KCNA2* expression and a reduction of total K_v_ current that was attributed to loss of K_v_1.2 channel function. Blockade of DNMT1 upregulation attenuated hyperexcitability in the injured DRG neurons and alleviated nerve injury-induced pain hypersensitivity (Sun et al., 2019).

A second a parallel pathway involving the MBD1 protein (Methyl-CpG-binding domain protein 1) has been described. MBD1 binds to methylated sequences of DNA and attracts the DNA methylation protein DNMT3a leading to downregulation of *KCNA2* and reduced K_v_1.2 expression. Overexpression of MBD1 leads to spontaneous pain and evoked pain hypersensitivities in wild type mice (Zhao et al., 2017; Mo et al., 2018).

Thirdly, SNL downregulates the expression of K_v_1.2 in DRG by decreasing expression of ten-eleven translocation methylcytosine dioxygenase 1 (TET1). This promotes DNA demethylation and its overexpression in the DRG of nerve injured animals alleviated pain hypersensitivities without altering acute pain. Mechanistically, TET1 rescued the expression of K_v_1.2 by reducing the level of 5-methylcytosine and increasing the level of 5-hydroxymethylcytosine in the promoter region of the *KCNA2* gene (Wu et al., 2019).

A separate mechanism involving histone deacetylase2 (HDAC2) is also thought to control K_v_1.2 expression in large DRG neurons in response to CCI (Li Z. et al., 2019).

There is also evidence that K_v_1.2 function is controlled by the non-coding miniature RNA miR-137. Because it impairs K_v_1.2 function, experimental impairment of miR-137 function, rescues channel expression and function attenuates allodynia in rats subject to CCI (Zhang et al., 2020).

Lastly, a long non-coding RNA contributes to neuropathic pain by silencing *KCNA2* and thereby reducing expression of K_v_1.2 in primary afferents (Zhao et al., 2013). This is of particular interest because K_v_1.1–K_v_1.2 heteromers function as mechanosensitive K^+^ currents that act as mechanical brake in the senses of touch and pain (Hao et al., 2013).

Interfering with K_v_1.1 channel expression results in mechanical allodynia without a change in thermal sensitivity. A role of K_v_1.1 channels in suppressing mechanical allodynia fits with their high expression in high threshold C-mechanoreceptors. These findings do not exactly correspond with the effect of CCI on TRPM8 expressing DRG neurons where there is loss of function of the “excitability brake current”. This current, which is thought to involve K_v_1.1 and 1.2 channels, normally restricts the depolarizing influence of TRPM8 channel activation by cold. It has been proposed that the loss of K_v_1.1/1.2 may thus promote cold allodynia (Gonzalez et al., 2017).

It is also possible that injury-induced changes in delayed rectifier channels reflect post-translational processes such as phosphorylation, endocytosis and/or trafficking (Nesti et al., 2004; Yang et al., 2007) that may be independent of any change in expression of K^+^ channel genes. This possibility is underlined by the observation that delayed rectifier currents were markedly reduced in medium- and large-, but not in small diameter DRG neurons in a rodent model of painful diabetic neuropathy but the mRNA levels for K_v_1.1, K_v_1.2, K_v_2.1, and K_v_2.2 were unchanged (Cao et al., 2010). mRNA for K_v_1.2 was, however, downregulated in the lumbar (L5) spinal nerve ligation (SNL)model (Fan et al., 2014). This illustrates the point made above that responses of the sensory system to injury are contingent on the nature of the injury (see Tables 1, 2).

Studies of the silent subunit K_v_9.1, have brought forth yet another mechanism whereby injury can influence delayed rectifier channels (Tsantoulas et al., 2012, 2018). K_v_9.1 forms heterotetramers with K_v_2.1 and K_v_2.2 (Kerschensteiner et al., 2005) and this interaction leads to alteration of the functional channel kinetics. SNI has been shown to downregulate K_v_9.1 in large DRG neurons and this may alter behavior of K_v_9.1 ∼ K_v_2.1. ∼ K_v_2.2 heterotetramers (Tsantoulas et al., 2012). Genetic modulation of K_v_9.1 expression *in vivo* produces changes in pain behavior consistent with its role in onset of neuropathic pain (Tsantoulas et al., 2012, 2018).

Since loss of delayed rectifier function augments nociceptive processes, restoration of function may lead to novel therapeutic approaches. Although preclinical studies have identified some substances that activate members of the delayed rectifier K^+^ channel family, for example RE1 and EX15 or AUT1 and AUT2 for K_v_3.1 (Taskin et al., 2015; Brown et al., 2016), glycine derivatives for K_v_1.1 (Manville and Abbott, 2020) and hanatoxin (from tarantula spiders) for K_v_2.1 (Milescu et al., 2013), any therapeutic potential for pain management remains to be established. On the one hand, some of these substances seem highly selective for the cogent channel subtypes but on the other hand, the wide distribution of delayed rectifier channels in both excitable and non-excitable tissues may limit their therapeutic effectiveness.

### Distribution of KCNQ/K_v_7.2/7.3/ M-Channels in Primary Afferent Neurons and Effects of Nerve Injury

These channels fall into the broad category of delayed rectifiers but are frequently discussed separately as they display limited structural similarity to other K_v_ channel types (Gutman et al., 2005). Unlike some other K_v_ channels, M-channels are modulated by G-protein coupled agonists (Selyanko et al., 1990; Suh et al., 2004), inflammatory mediators (Linley et al., 2008), including bradykinin (Cruzblanca et al., 1998) and ATP (Ford et al., 2003), src-tyrosine kinases (Gamper et al., 2003), Ca^2+^ acting via calmodulin (Gamper and Shapiro, 2003; Li et al., 2005) and by the membrane phospholipid phosphatidylinositol 4,5-bisphosphate (PIP_2_) (Ford et al., 2003; Li et al., 2005; Du et al., 2018). G_q_ coupled agonists suppress M-channel conductance via phospholipase C mediated depletion of PIP_2_ (Ford et al., 2003; Suh et al., 2004). M-channels are opened by the anti-epileptic drug, retigabine (Brown and Passmore, 2009).

K_v_7.1 is derived from the *KCNQ1* gene and is confined to non-neuronal tissue. It plays an important role in the repolarization of cardiac action potentials. By contrast K_v_7.2, 7.3, 7.4, and 7.5 which derive from the *KCNQ2, 3, 4*, and *5* genes are predominantly neuronal (Gutman et al., 2005; Brown and Passmore, 2009). Classical M-channels are hetero-tetramers comprised of K_v_7.2 and 7.3 (Shapiro et al., 2000; Hadley et al., 2003) which show maximal activation at −30 mV. Because of this, they exert profound attenuation of repetitive action potential discharge in several neuron types (Adams et al., 1982; Cooper et al., 2001; Brown and Passmore, 2009) including nociceptors (Barkai et al., 2017; Du et al., 2018).

Pharmacologically identified M-currents and the relevant mRNA’s have been detected in cultured rat DRG neurons (Passmore et al., 2003; Rose et al., 2011). K_v_7.2 is more abundant in small and medium sized neurons than in large neurons (Rose et al., 2011; Du et al., 2018). In addition to their role in controlling excitability of DRG cell bodies, modeling studies have identified the importance of M-channels in the regulation of action potential propagation from the periphery, past the DRG to the CNS (Sundt et al., 2015; Du et al., 2018) as well as their role in controlling the excitability of primary afferent terminals in the dorsal horn (Barkai et al., 2017).

Decreases in expression of KCNQ2 and/or KCNQ3 proteins and a reduction of M-current density in the cell bodies of small-sized DRG neurons have been reported in CCI (Rose et al., 2011) and bone cancer pain (Zheng et al., 2013). Moreover, impairment of expression of K_v_7.2 induces thermal and mechanical hyperalgesia in naïve animals (King et al., 2014). In line with this, the K_v_7 activator, retigabine has been shown to alleviate neuropathic pain behavior in a rodent model of diabetic neuropathy (Djouhri et al., 2019). The effectiveness of retigabine paralleled that of gabapentin and its effect was reversed by the K_v_7/M-channel blocker, XE991. Although some of the anti-nociceptive effectiveness of the COX2 inhibitor, celecoxib has been attributed to an ability to activate K_v_7.2/7.3 channels expressed in HEK293 cells and M-type current in DRG neurons (Mi et al., 2013), this may have little or no therapeutic implication as NSAIDs generally display little or no effectiveness in the management of neuropathic pain (Yekkirala et al., 2017).

Considerable progress has been made in understanding the molecular machinery associated with down-regulation of the expression of *KCNQ2* and *3* in DRG in response to peripheral nerve injury. *KCNQ* genes have functional repressor element 1 (NRSE) binding sites (Mucha et al., 2010) and the effects of partial sciatic nerve ligation depend on repressor element 1-silencing transcription factor (REST, also known as NRSF) (Mucha et al., 2010; Rose et al., 2011). Viral overexpression of REST in DRG neurons strongly suppresses M-current density and increases excitability (Mucha et al., 2010). REST inhibits transcription by recruiting the co-repressor complexes SIN3A/B and REST corepressor 1; these complexes, in turn, modify target gene regions through the action of HDAC1/2, the histone demethylase LSD1 and the histone methylase G9a (Ooi and Wood, 2007; Willis et al., 2016).

Inhibition or genetic deletion of G9a in DRG abolished injury-induced down-regulation of K_v_7.2 and reduced neuropathic hyperalgesia. G9a may have an important role in K^+^ channel regulation as it has also been implicated in injury induced suppression of K_v_1.4, K_v_4.2 and BK channels (K_Ca_1.1) (Laumet et al., 2015).

The effectiveness of retigabine may not only reflect reduced excitability of DRG cell bodies (Passmore et al., 2003), but may also involve reduced excitability of C-fiber axons (Lang et al., 2008). As mentioned above, this may be especially important for propagation of action potentials in unmyelinated fibers adjacent to DRG cell bodies, where the safety factor for transmission is low. Transmission is attenuated in a model where a KCNQ component is included the soma, stem axon, and proximal (to the T-junction), peripheral and central axons (Sundt et al., 2015). Another modeling study underlined the possible role of K_v_7.2/7.3 channels in controlling the activity of primary afferent terminals in the dorsal horn (Barkai et al., 2017). Additional sites where K_v_7.2/7.3 channels influence sensory nerve activity include the neuroma generated by injury to peripheral nerves (Cisneros et al., 2015; Bernal et al., 2016) as well as intact Aδ mechanoreceptor terminals in the skin (Schutze et al., 2016).

Taken together, these findings underline the importance of K_v_7.2/7.3 channels in controlling sensory neuron excitability and outline their potential as a therapeutic target in the management of neuropathic pain (Rivera-Arconada et al., 2009; Barrese et al., 2018). Unfortunately, a clinical study of retigabine in human neuropathic pain (post herpetic neuralgia) failed to meet its efficacy endpoint (Yekkirala et al., 2017). Similarly flupirtine which is chemically related to retigabine (Abd-Elsayed et al., 2019; Osuma et al., 2019; Surur et al., 2019; Shi et al., 2020) and which was used as a non-opioid analgesic in Europe for many years, showed promise as an anti-allodynic agent. Unfortunately, this drug has now been withdrawn for all indications owing to its propensity to cause hepatotoxicity. Despite these setbacks at least 200 K_v_7.2 activators are currently under development (Du and Gamper, 2013; Yekkirala et al., 2017). It is interesting to note in this regard that the frequently prescribed anti-allodynic agent gabapentin, which is thought to only interact with Ca^2+^ channels (Field et al., 2006), has been recently been reported to activate K_v_7.3 and 7.5 channels when they are expressed in *Xenopus* oocytes (Manville and Abbott, 2018).

### A-Channels

Tetrameric, rapidly activating, inactivating, voltage-gated K^+^ currents assemble from K_v_1.4, 3.3, 3.4, 4.1, 4.2, and 4.3 subunits that are products of the *KCNA4, KCNC3, KCCN4, KCND1, KCND2*, and *KCND3* genes (Gutman et al., 2005). Such channels are widely distributed in the cell bodies of DRG neurons (McFarlane and Cooper, 1991; Gold et al., 1996a; Safronov et al., 1996; Everill et al., 1998; Abdulla and Smith, 2001b; Matsuyoshi et al., 2012; Yunoki et al., 2014; Stemkowski et al., 2015) and display a considerable range of biophysical and pharmacological properties. For example, τ for inactivation can very between 4 and 190 ms at +50 or +60 mV and τ for recovery from inactivation can range between 2000 and 43 ms at −100 mV. Although this reflects classical N-type “ball and chain” inactivation in K_v_1.4 and K_v_3.4 channels (Kanda et al., 2011), the mechanism of inactivation of K_v_4.1, 4.2 and 4.3 is not fully understood. Whereas K_v_3.4 channels are sensitive to sub-millimolar concentrations of 4-aminopyridine (4-AP) somewhat higher concentrations are required to block K_v_4.1, 4.2, and 4.3 (Zemel et al., 2018).

#### Distribution of A-Type K_v_ Channels Exhibiting N-Type Inactivation (K_v_1.4) in Primary Afferent Neurons and Effects of Nerve Injury

K_v_1.4 channels are especially abundant in small diameter IB4-positive DRG neurons (Vydyanathan et al., 2005) where their function and surface expression is modulated by phosphorylation, growth factors, inflammatory mediators and auxiliary subunits (Takeda et al., 2008; Zhu et al., 2012a,b; Zemel et al., 2018). Most manipulations capable of producing painful neuropathy attenuate K_v_1.4 expression and/or function in DRG neurons (Kim et al., 2002).

In a bone cancer model, however, there are time-dependent increases in A-type K_v_ channels that are expressed in IB4-positive, small DRG neurons. Currents are increased on post-tumor day 14 but then diminish yet remain at a higher level than control for an additional 7 days. The expression of K_v_1.4 protein quantified by immunohistochemistry (IHC) and Western immunoblots (WB), as well as that for K_v_3.4, and K_v_4.3, show corresponding time-dependent increases (Duan et al., 2012). There is also evidence that injury-induced down regulation of delayed rectifier K_v_1.1. and K_v_1.2 at the neuroma that forms after nerve injury is balanced by increased expression of K_v_1.4 and 1.6 (Calvo et al., 2016).

This time-dependent replacement of one channel type by another may relate to the partial recovery of mechanical hypersensitivity seen in some nerve injury models such as CCI but not after SNI (Decosterd and Woolf, 2000; Noh et al., 2020).

#### Distribution of High Threshold, N-Type Inactivating, 4-AP and TEA Sensitive A-Currents (K_v_3.4) and Effects of Nerve Injury

Although the axon, soma and spinal terminals of all sensory neurons express K_v_3.4, expression is especially strong in small diameter neurons (Ritter et al., 2012, 2015a,b; Muqeem et al., 2018; Zemel et al., 2018). K_v_3.4 likely corresponds to high threshold A-currents first described by Gold et al. (1996a) and since these currents were found in capsaicin sensitive neurons, K_v_3.4 channels may be strongly associated with nociceptors (Zemel et al., 2018); a prediction confirmed by immunohistochemical (IHC) studies (Chien et al., 2007). K_v_3.4 in nociceptors contributes 40-70% of the total repolarizing charge during the AP (Ritter et al., 2015b). These channels are especially sensitive to 4-aminopyridine (4-AP) and tetraethylammonium (TEA) block and are regulated by oxidation, phosphorylation and ancillary proteins (Zemel et al., 2018).

Dysfunction in K_v_3.4 channels has been described in several chronic pain models and additional data underline their role in pain etiology. Thus, sciatic nerve ligation has been shown to reduce K_v_3.4 immunoreactivity in the cell bodies, peripheral axons and central terminals of primary afferent nociceptors (Chien et al., 2007) and suppression of K_v_3.4 expression following intrathecal injection of antisense oligodeoxynucleotides into uninjured animals produces mechanical hypersensitivity (Chien et al., 2007). Similar results were obtained in a model of diabetic neuropathy, the densities of total A-currents were markedly reduced in medium- and large-, but not in small-diameter DRG neurons and this was matched by changes in mRNA levels for K_v_1.4, K_v_3.4, K_v_4.2, and K_v_4.3 (Cao et al., 2010).

Muqeem et al. (2018) took advantage of the high sensitivity of K_v_3.4 channels to 4-AP and TEA to demonstrate that channel blockade increased monosynaptic excitatory postsynaptic currents (EPSCs) in dorsal horn laminae I and II neurons through a presynaptic mechanism. K_v_3.4 function is impaired in primary afferents after nerve injury (Chien et al., 2007; Cao et al., 2010; Duan et al., 2012) and excitatory spontaneous synaptic activity is increased in excitatory lamina II neurons (Balasubramanyan et al., 2006; Chen et al., 2009b). These findings taken together underline the importance of K_v_3.4 in primary afferent terminals in central sensitization and pain.

Lastly, and already alluded to above (section “Spinal Cord Injury and Changes in Peripheral Neuron Excitability”) K_v_3.4 current amplitude, inactivation, and channel membrane expression are reduced in the DRG following unilateral spinal cord contusion (Ritter et al., 2015a; Zemel et al., 2017). Since total K_v_3.4 protein and mRNA in the DRG did not change, these results suggest a post-translational effect (Ritter et al., 2015b; Zemel et al., 2018) possibly involving dysregulation of calcineurin (Zemel et al., 2017).

#### Distribution and Regulation of K_v_4.1, 4.2, and 4.3 A-Type K_v_ Channels in Primary Afferent Neurons and Effects of Nerve Injury

Immunoreactivity and/or mRNA for K_v_4 type channels is found in the cell bodies of small and large DRG neurons (Kim et al., 2002). K_v_4.3 appears selectively in the cell bodies of a subset of non-peptidergic DRG neurons (Chien et al., 2007; Yunoki et al., 2014; Kuo et al., 2017) whereas K_v_.4.1 is expressed in all sizes of DRG neurons (Matsuyoshi et al., 2012; Yunoki et al., 2014). Electrophysiological and molecular studies and the use of a K_v_4-specific dominant negative probe implicate K_v_4.1 and 4.3 as the molecular correlate of subthreshold A-currents in DRG neurons (Phuket and Covarrubias, 2009).

The function and expression of K_v_4 channels in the DRG is controlled by signaling pathways such as MAPK (Grabauskas et al., 2011), accessory subunits such as K_v_4 channel interacting proteins (KChIPs) and dipeptidyl-peptidase-like proteins (DPPLs) (Amarillo et al., 2008; Jerng et al., 2009; Pongs and Schwarz, 2010) as well as transcription factors such as the neuron restrictor silencer factor (REST), which suppresses transcription of the K_v_4.3 gene (*KCND3*) after nerve injury (Uchida et al., 2010b).

The downregulation of mRNA for K_v_4.2/4.3 channels and/or their surface expression and/or I_A_ occurs in DRG neurons in a variety of nerve injury models, thereby implicating K_v_4 dysfunction in chronic neuropathic pain (Chien et al., 2007; Cao et al., 2010; Grabauskas et al., 2011; Kuo et al., 2017; Zemel et al., 2018).

The modulatory subunits KChIP1, KChIP2, and DPP10 form a K_v_4.3/KChIP1/KChIP2/DPP10 complex in DRG neurons. Knockdown of the expression of any component of this complex promotes mechanical hypersensitivity and increased excitability of non-peptidergic nociceptors. Spinal nerve ligation downregulates the expression of all K_v_4 complex components but this is rescued using cDNA constructs encoding K_v_4.3, KChIP1, and DPP10. This is accompanied by attenuation of SNL-induced mechanical hypersensitivity and partial recovery of K_v_4.3, KChIP1, and DPP10 surface levels in the injured DRGs. This demonstrates that the K^+^ channel modulatory subunits KChIP1, KChIP2, and DPP10 participate in K_v_4.3-mediated mechanical pain control (Kuo et al., 2017).

Although a few compounds have been identified which directly open delayed rectifier K^+^ channels by altering their biophysical properties (Milescu et al., 2013; Taskin et al., 2015; Brown et al., 2016; Manville and Abbott, 2020) less progress has been made in identifying direct activators of A-channels. The compound KW-7158 suppresses afferent nerve activity as a result of its ability to potentiate I_A_ in DRG neurons (Sculptoreanu et al., 2004). Although KW-7158 was proposed as a treatment for overactive urinary bladder (Maeda et al., 2012), any efficacy of this compound in pain models remains to be established.

Drugs which target accessory subunits of A-channels may provide an alternative strategy (Zemel et al., 2018). DPPLs and KChIPs not only govern the biophysical properties of K_v_ channels. They also impact channel assembly, channel trafficking to and from the cellular surface, and targeting of channels to different cellular compartments (Pongs and Schwarz, 2010). The observation that knockdown of any component of the K_v_4.3/KChIP1/KChIP2/DPP10 complex promotes mechanical hyperalgesia has already been alluded to (Kuo et al., 2017).

Lastly, targeting signaling pathways and epigenetic mechanisms that control expression, trafficking and properties of A-type channels may provide the theoretical basis for novel approaches to chronic pain management. Two types of mechanism may be involved,

(1) Nerve injury upregulates a micro-RNA cluster (mir-17-92) in rat DRG neurons and its overexpression reduces the expression of A-type K^+^ channels and some of their accessory subunits. Overexpression of certain members of this cluster elicited mechanical allodynia and specific blockade of micro-RNA function with antisense oligomers alleviated pain in nerve injury models (Sakai et al., 2017). The therapeutic potential of antisense targeting of mir- 17-92 or other micro RNAs remains to be exploited.
(2) Nerve injury increases activity of the histone-lysine *N*-methyltransferase 2 (G9a). This enzyme regulates euchromatic gene expression via histone modification. This in turn affects the promoters for the A-channel genes *KCNA4* (K_v_1.4) and *KCND2* (K_v_3.2), as well as that for (K_v_7.2) *KCNQ2* and *KCNMA1* [for the BK channel K_Ca_1.1, see sections “Distribution of KCNQ/K_v_7.2/7.3/M-channels in Primary Afferent Neurons and Effects of Nerve Injury” and “Distribution of BK or Maxi K^+^ Channels (K_Ca_1.1) in Primary Afferent Neurons and Effects of Nerve Injury”]. G9a inhibition or ablation restored K^+^ channel expression in the DRG and attenuated pain hypersensitivity. Interestingly inhibition of G9a but also normalized the expression of many other genes altered by nerve injury (Laumet et al., 2015). These findings implicate G9a in the transcriptional silencing associated with neuroplasticity in neuropathic pain. While there are some reports of the efficacy of small molecule G9a inhibitors in rodent models of neuropathic pain (Wang et al., 2017; Liang et al., 2019) any therapeutic potential of these substances is yet to be explored.

### Calcium (and Sodium) Activated K^+^ Channels

Ca^2+^ activated K^+^ channels and their corresponding conductances (g_*K,Ca*_) are divided into two broad categories on the basis of low or high single channel conductance (Wei et al., 2005; Berkefeld et al., 2010). The first group are voltage-insensitive and activated by low concentrations of intracellular Ca^2+^ (<1.0 μM). Ca^2+^ acts via binding to calmodulin which is tightly complexed to the C-terminal of the channel protein (Berkefeld et al., 2010). This group includes the small conductance K_Ca_2.1, 2.2, and 2.3 channels (SK_Ca_1, SK_Ca_2, and SK_Ca_3) which are products of the *KCNN1, 2* and *3* genes and which are blocked by 100 pM–10 nM apamin (Wei et al., 2005). Single channel conductance for this group of channels is <10ps. The grouping also includes the apamin-insensitive, clotrimazole-sensitive intermediate conductance channel (K_Ca_ 3.1 encoded by *KCNN4*, single channel conductance 11pS; also known as the I_AHP_ or IK_Ca_1channel).

The second group includes the K_Ca_1.1 (Slo or Slo1), K_Ca_4.1 (Slack or Slo 2.2), K_Ca_4.2 (Slick or Slo 2.1) and K_Ca_5.1 (Slo3) channels which are products of the *KCNMA1, KCNT1, KCNT2* and *KCNU1* genes respectively. K_Ca_1.1 is regulated by four different β-subunits encoded by the *KCNMB1-4* genes. It is also known as the “maxi K^+^ channel,” the BK channel or the BK(Ca) channel by virtue of its high conductance (260pS). Its activation requires both depolarization and the direct interaction of Ca^2+^ with the channel. K_Ca_1.1 is blocked by low concentrations of TEA (0.14 mM), and by charybdotoxin (2.9 nM) or iberiotoxin (1.7 nM). Channels of this type are rapidly activating and intimately associated with voltage gated Ca^2+^ channels (Robitaille et al., 1993; Jassar et al., 1994; Berkefeld et al., 2010; Zhang et al., 2018). They thus serve as a powerful braking mechanism for voltage-gated Ca^2+^ influx. This has obvious implications for the control of neurotransmitter release (Furukawa et al., 2008; Hoppa et al., 2014). The tight association of K_Ca_1.1 with Ca^2+^ channels means that their activity is readily attenuated by divalent cations such as Cd^2+^ which block closely associated voltage-gated Ca^2+^ channels (Abdulla and Smith, 2001b).

The inclusion of K_Ca_4.1 (Slack or Slo 2.2) and K_Ca_4.2 (Slick or Slo 2.1) in this grouping is based on their structural similarity to K_Ca_1.1 (Wei et al., 2005) but this is somewhat misleading in practical terms as they display little sensitivity to Ca^2+^ and are more readily activated by internal Na^+^ and Cl^–^ (Yuan et al., 2003). They are known as Na^+^ activated K^+^ channels and require both depolarization and internal Na^+^ to activate. The category also includes the pH sensitive high conductance K^+^ channel K_Ca_5.1 which is encoded by the *KCNMC1* gene (Wei et al., 2005).

#### Distribution of Small Conductance K_Ca_ Channels (K_Ca_2.1, 2.2, 2.3) in Primary Afferent Neurons and Effects of Nerve Injury

In rodents, K_Ca_2.1, 2.2, and 2.3 have been localized to IB4-positive and to peptidergic nociceptors (Mongan et al., 2005) and these express an apamin-sensitive g_K,Ca_ which is reduced by SNL (Sarantopoulos et al., 2007). This correlates with the clinical postmortum immunohistochemical (IHC) studies suggest that K_Ca_2.1 is decreased in DRG avulsed from neuropathic pain patients (Boettger et al., 2002).

Positive modulators of K_Ca_2 and K_Ca_3 channels, 4,5-dichloro-1,3-diethyl-1,3-dihydro-benzoimidazol-2-one (NS4591) and 4-(2-methoxyphenylcarbamoyloxymethyl)-piperidine-1-carboxylic acid tert-butyl ester (GW542573X) have been identified (Hougaard et al., 2009a,b) and their possible effectiveness in neuropathic pain is yet to be explored.

#### Distribution of Intermediate Conductance K_Ca_ Channels (K_Ca_3.1) in Primary Afferent Neurons and Effects of Nerve Injury

Although the work of Boettger et al. (2002) and Sarantopoulos et al. (2007) also reported decreased function of K_Ca_3.1 in DRG following nerve injury, the relevance of these findings to pain etiology is questioned by the observation that K_Ca_3.1-/1 mice show normal behavioral responses in models of neuropathic pain (Lu et al., 2017).

#### Distribution of BK or Maxi K^+^ Channels (K_Ca_1.1) in Primary Afferent Neurons and Effects of Nerve Injury

Iberiotoxin or charybdotoxin sensitive BK channels (K_Ca_1.1) are present in all DRG neurons (Abdulla and Smith, 1999; Li et al., 2007; Zhang et al., 2010; Cao et al., 2012). They are especially enriched in the IB4-positive population of small neurons (Zhang et al., 2010).

Sciatic nerve section reduces overall Cd^2+^-sensitive Ca^2+^ sensitive K^+^ conductance in small, medium and large sized DRG neurons (Abdulla and Smith, 2001b). Although these changes were attributed to suppression of the associated voltage gated Ca^2+^ channels, more recent work has underlined the direct attenuation of K_Ca_ channel function (Sarantopoulos et al., 2007). This study used the SNL model and while BK conductance decreased in those medium sized neurons where axons were actually severed (axotomized L5 DRG), BK channel function was increased in adjacent undamaged neurons (L4 DRG). By contrast, in small neurons BK current was reduced in both axotomized (L5) and adjacent, undamaged (L4) neurons. SNL induced decrease in BK function in small and medium DRG neurons has been confirmed by others (Cao et al., 2012) but these authors did not address the possibility that axotomized and undamaged neurons respond differently in response to SNL.

SNL downregulates *KCNMA1* in whole DRG as a result of G9a activation (Laumet et al., 2015) and the protein levels of BK channels are substantially reduced in small- and medium-sized DRG neurons (Chen et al., 2009a). These authors showed that blocking the BK channel with iberiotoxin reduced the mechanical withdrawal threshold in control and nerve-injured rats. Intrathecal injection of the BK channel opener [1,3-dihydro-1-[2-hydroxy-5-(trifluoromethyl)phenyl]-5-(trifluoromethyl)-2H-benzi midazol-2-one] reversed allodynia and hyperalgesia in nerve-injured rats but it had no significant effect on nociception in control rats.

SNI is known to increase the frequency of spontaneous excitatory postsynaptic currents (sEPSCs) in neurons in the rat superficial dorsal horn (Balasubramanyan et al., 2006; Chen et al., 2009b). In mice, iberiotoxin increased the frequency of sEPSCs in control mice to the same level as that in the nerve injured mice but did not increase sEPSC frequency in nerve injured mice. These findings support the possibility that BK channels located in presynaptic terminals control synaptic transmission in the superficial dorsal horn, and that functional downregulation of BK channels contributes to the central sensitization that characterizes neuropathic pain (Furukawa et al., 2008). In addition to this, it has been reported that activation of BK in DRG cell bodies can impede passage of impulses through the T-junction on the primary afferent fiber, effectively acting as a low pass filter for propagation of action potentials towards the CNS (Gemes et al., 2013). When BK channels are impaired, after spinal nerve ligation (SNL), a higher frequency of action potentials can pass. This too may contribute to increased frequency of sEPSC’s recorded in the spinal cord.

Given the established role of BK in determination of spike width in DRG (Scholz et al., 1998; Zhang X. L. et al., 2012; Noh et al., 2019) and action potential propagation (Gemes et al., 2013), the implications for increased neurotransmitter release in the spinal dorsal horn (Furukawa et al., 2008), a recent report that overexpression of BK increased mechanical threshold in a rodent neuropathic pain model (Zhang et al., 2018), and the availability of a BK activator (Chen et al., 2009a), these channels would seem at least as viable a target as K_v_7 and A-channels for therapeutic intervention.

The work by Zhang et al. (2018) also brought forth an alternative mechanism whereby altered BK expression could relate to neuropathic pain mechanisms. It is well established that the cell surface expression and trafficking of Ca_v_2.2 (N-type Ca^2+^ channels) is controlled by the α2δ1 accessory subunit (Dolphin, 2016) and these are upregulated in DRG after peripheral nerve injury (Hoppa et al., 2012). Injury would thus lead to increased abundance of Ca_v_2.2 channels in primary afferent terminals and increased transmitter release. Because BK channels were shown to also bind to α2δ1, it has been suggested they compete with Ca_v_2.2, thus normally restricting surface expression of Ca_v_2.2 (Zhang et al., 2018). Following downregulation of BK by injury (Cao et al., 2012), α2δ1 would be released to interact with Ca_v_2.2 and increase their surface expression and to increase transmitter from primary afferent terminals.

It has also been suggested that endoplasmic reticulum stress associated with nerve injury may contribute to pain by alteration of BK function (Yousuf et al., 2020).

Several positive modulators of BK function have been identified (Chen et al., 2009a; Hoshi and Heinemann, 2016), including the highly effective GoSlo-SR family of anthraquinone analogs (Roy et al., 2012) but these have not yet been evaluated as possible anti-allodynic agents. On the other hand, there is considerable discussion in the literature relating to the efficacy of cannabinoids in neuropathic pain (Cristino et al., 2020) and it has been suggested that augmentation of BK function may contribute to their therapeutic effect (Li Y. et al., 2019).

#### Distribution of Na^+^-Sensitive K^+^ Channels (Slack and Slick; K_Ca_4.1 and 4.2) in Primary Afferent Neurons

An Na^+^ sensitive K^+^ conductance was first described in small DRG neurons and myelinated sensory axons over 20 years ago (Poulter et al., 1995; Bischoff et al., 1998). Single channel activity of K_Ca_4.1 in IB4-positive nociceptors is controlled by the anoctamin protein TMEM16C. TMEM16C knockout rats or those in which slack (K_Ca_4.1) is knocked down using siRNA exhibit increased thermal and mechanical sensitivity (Huang et al., 2013). Similarly, global or selective ablation of slack (K_Ca_4.1) in DRG led to increased hypersensitivity in neuropathic pain models whereas inflammatory and acute nociceptive pain were unaffected (Lu et al., 2015). These effects may be mediated via slack channels (K_Ca_4.1) regulation of neurotransmitter release from primary afferent terminals (Evely et al., 2017). The atypical antipsychotic drug, loxapine which has been reported to activate K_Ca_4.1/slack (Biton et al., 2012), ameliorated persisting neuropathic pain behaviors in the rat SNI model (Lu et al., 2015). Unfortunately a pilot study of loxapine effectiveness in chronic pain patients had to be terminated due to adverse events seen in all patients involved in the study (Schmiedl et al., 2019).

### Two Transmembrane-Domain, Inwardly Rectifying K^+^ Channels (K_IR_)

Inwardly rectifying channels are opened by membrane hyperpolarization and favor inward flux over outward flux of K^+^ ions. There are seven subfamilies of K_IR_ channels, denoted as K_IR_1 – K_IR_7 (Kubo et al., 2005). Each functional channel contains four subunits each with two transmembrane domains.

Instantaneous inward rectifiers, which among other processes, are involved in repolarization of the cardiac Purkinje fiber action potential include K_IR_1.1, 2.1, 2.2, 2.3, and 2.4. These are the respective products of the *KCNJ1, KCNJ2, KCNJ12, KCNJ4*, and *KCNJ14* genes.

K_IR_3.1, 3.2, 3.3, and 3.4 are also known as G-protein coupled inwardly rectifying K^+^ channels GIRK 1-4. They are activated by acetylcholine in the heart and by a variety of G_i_ coupled agonists throughout the nervous system. They are products of the *KCNJ3, KCNJ6, KCNJ9*, and *KCNJ5* genes.

K_IR_6.1 and 6.2 channels which are products of *KCNJ8* and *KCNJ11* genes are controlled by the intracellular ATP to ADP ratio. K_IR_6.2 [also known as I_K(ATP)_] is involved in insulin secretion from pancreatic islet cells. It is also found in neurons and other excitable cells.

K_IR_4.1 channels are found primarily in glial cells and have important roles in brain K^+^ buffering. K_IR_4.2 and Kir 5.1channels are largely non-neuronal in distribution and K_IR_7.1 is of particular importance pigmented epithelial cells of the retina (Kubo et al., 2005; Kumar and Pattnaik, 2014).

#### Distribution of Instantaneous Inwardly Rectifying K^+^ Channels K_IR_1.1, 2.1, 2.2, 2.3, and 2.4) in Primary Afferent Neurons

An instantaneous, Ba^2+–^-sensitive inwardly rectifying K^+^ current has been described in medium sized DRG neurons (Scroggs et al., 1994). In agreement with this, confocal imaging revealed K_IR_2.1, K_IR_2.2, and K_IR_2.3 immunoreactivity in most DRG neurons as well as in nerve terminals in spinal lamina II (*substantia gelatinosa*). K_IR_2.3 was also seen in satellite glial cells and all three K_IR_ channels were found in spinal astrocytes (Murata et al., 2016). Any effects of nerve injury on these currents remains to be explored.

#### Distribution of GIRK (K_IR_3.1, 3.2, 3.3, and 3.4) Channels in Primary Afferent Neurons and Effect of Nerve Injury

Application of G_i_ coupled agonists such as μ-opioids or α2 adrenergic suppress N-type Ca^2+^ currents and open GIRK channels in the cell bodies of DRG neurons (Abdulla and Smith, 1997, 1998; Nockemann et al., 2013; Stotzner et al., 2018). Complementary studies reported the presence of GIRK1/K_IR_3.1 immunoreactivity in 70% of DRG neurons and although GIRK2/K_IR_3.2 only appears in 10% of neurons, these appear to be small peptidergic nociceptors. These findings are not in complete agreement with those of Nockemann et al. (2013) who described the presence of both GIRK1/Kir3.1 and GIRK2/Kir3.2 in IB4 positive non-peptidergic neurons. GIRK channels may be transported to sensory nerve endings in the skin (Nockemann et al., 2013) and to presynaptic terminals in the spinal cord, particularly lamina II (Lyu et al., 2015). Opioids and other G_i_ coupled agonists are well known to attenuate neurotransmitter release from primary afferents (Kohno et al., 1999) and this is thought to involve an action on Ca^2+^ channels (Heinke et al., 2011). Since opioid actions are not blocked by the GIRK channel blockers Ba^2+^ and Cs^+^, it is unlikely that they are involved in this process (Heinke et al., 2011). Despite this, there is good evidence to suggest a role for GIRK2/K_IR_ 3.2 channels in sensory nerve endings in the actions of peripherally acting opioids (Nockemann et al., 2013). Although only 18% of DRG cell bodies display immunoreactivity for K_IR_3.3, channel protein is present in nerve endings in glabrous skin and may be present in primary afferent terminals in the spinal cord (Lyu et al., 2020).

Nerve injury down-regulates GIRK1/K_IR_3.1 in DRG neuron cell bodies at the mRNA and protein levels and reduces its expression in the spinal dorsal horn (Lyu et al., 2015). Despite the reported presence of functional K_IR_ channels in DRG cell bodies (Stotzner et al., 2018) altered expression may only affect DRG excitability in the presence of appropriate G_i_ coupled ligands. It is well known, however, that opioid effectiveness is limited in neuropathic pain states (Abdulla and Smith, 1998; Sun et al., 2017) and this may involve a decrease in their effectiveness in limiting transmitter release from primary afferents terminals (Kohno et al., 2005). It remains to be determined whether this effect is contingent upon down regulation of K_IR_ as reported by Lyu et al. (2015).

#### Distribution of ATP Sensitive K^+^ (K_IR_6.1 and 6.2) Channels in Primary Afferent Neurons and Effect of Nerve Injury

By contrast with the limited information and possible limited significance of effects of nerve injury on instantaneous inward rectifiers and GIRK channels in primary afferents, somewhat more information is available regarding K_IR_6.1 and 6.2; the K_ATP_ channels (Ocana et al., 2004). These channel proteins co-assemble with sulphonylurea receptors (SUR or ATP binding cassettes) (Campbell et al., 2003). SUR are the molecular targets of the sulphonylurea drugs such as glibenclamide and tolbutamide which are used in the treatment of diabetes. Channel activation can be achieved by pinacidil and by the anti-hypertensive agent, diazoxide. The association of four K_IR_6 and four SUR subunits form a functional K_ATP_ channel. These channel complexes are activated by ADP and inhibited by ATP (Kubo et al., 2005).

Immunoreactivity for SUR1, SUR2, and Kir6.2 has been detected in DRG (Kawano et al., 2009). This observation was confirmed by Western blots for Kir6.2/SUR1 and Kir6.2/SUR2 K_ATP_ channels (Zoga et al., 2010). Kawano et al. (2009) also recorded spontaneous activity of single K_ATP_ channels in cell-attached patches made from all subpopulations of DRG neuron. Higher open probabilities and longer open times were seen in large compared to small neurons. K_ATP_ activity was suppressed only in large neurons from hyperalgesic rats, but not from animals that did not develop hyperalgesia in response to spinal nerve ligation (SNL). Injury did not affect ATP sensitivity, inward rectification, unitary conductance or the pharmacological properties of K_ATP_ channels. Although the anti-nociceptive actions of K_ATP_ channel openers has been recognized for many years (Vergoni et al., 1992; Welch and Dunlow, 1993), much of the preclinical work has dealt with the ability of drugs such as pinacidil to potentiate opioid action (Vergoni et al., 1992; Fisher et al., 2019). The relevance of this to therapeutic management of neuropathic pain is yet to be explored.

### Distribution of Four Transmembrane-Domain Tandem Pore Domain (K_2p_) Channels in Primary Afferents and Effects of Nerve Injury

Four transmembrane-domain tandem pore domain (K_2p_) channels account for K^+^ leak conductance and set the resting membrane potential of most excitable cells (Goldstein et al., 2005; Enyedi and Czirjak, 2010; Gada and Plant, 2019). They are different from other K^+^ channels as they assemble as dimers rather than tetramers and contain 2 pore domains.

At least 15 different types of K_2p_ channels have been identified that are products of corresponding *KCNK* genes. Channels are subcategorized as TWIK (Tandem of pore domains in a weak inwardly rectifying K^+^ channel), TREK (TWIK related K^+^ channel), TASK (TWIK related acid-sensing K^+^ channel), TRAAK (TWIK-related arachidonic acid activated K channel, TALK (TWIK related alkaline pH activated K^+^ channel), THIK (Tandem pore domain halothane inhibited K^+^ channel) and TRESK (TWIK related spinal cord K^+^ channel). This nomenclature has now been standardized so that 15 available *KCNK* genes correspond more exactly to the identity of the channels (i.e., K_2p_1-7, K_2p_9-10, K_2p_12-13, K_2p_15-18, Gada and Plant, 2019). In symmetrical K^+^ solutions, conductance of K_2p_ is instantaneous and ohmic whereas in physiological K^+^ solutions channels exhibit outward rectification as predicted by the Goldman–Hodgkin–Katz equation (Enyedi and Czirjak, 2010). With hindsight since the discovery of these channels (Ketchum et al., 1995), this means that most instantaneous current responses evoked by voltage commands involve K_2p_ channels.

mRNA coding for TASK-1 (*KCNK3*), TASK-2 (*KCNK5*), TASK-3 (*KCNK9*), TREK-1 (*KCNK2*), TREK-2 (*KCNK10*), TRAAK (*KCNK4*), TWIK-1 (*KCNK1*), and TRESK (*KCNK18*) has been found in rat and mouse DRG (Talley et al., 2001; Kang and Kim, 2006; Tulleuda et al., 2011; Marsh et al., 2012; Gada and Plant, 2019). These channels preferentially localize to small neurons (Gada and Plant, 2019). The superficial layers of spinal cord and small-diameter and/or or medium sized neurons of dorsal root ganglia also showed TASK-1, 2 or 3 (K_2p_3, 9 and 10) immunoreactivity (Gabriel et al., 2002; Rau et al., 2006) and presumptive and functional TASK-2 (k_2p_10) channels have also been recorded in cell attached patches on DRG neurons (La et al., 2006).

K_2p_ channels are highly regulated by a broad variety of intracellular and extracellular signals such as oxygen tension, pH, lipids, neurotransmitters, G-proteins, volatile and local anesthetics, temperature, kinases, cyclic AMP, small ubiquitin-related modifier proteins and scaffolding protein and their role in chronic inflammatory pain is well documented (Marsh et al., 2012). The mechanical and thermal sensitivity of K_2p_ channels is of special relevance as it has been suggested that TRAAK, TREK-1, and TREK2 are involved in polymodal pain perception (Kang et al., 2005; Alloui et al., 2006; Noel et al., 2009).

TRESK (k_2p_18) channels play an important role in the leak conductance of DRG neurons and their functional knockout produced a notable decrease in rheobase (Dobler et al., 2007). The relevance of this observation to neuropathic pain is supported by the observation that sciatic nerve transection reduces TRESK/(k_2p_18)/*KCNK18* mRNA in DRG to a greater extent than other K_2p_ channels and *in vivo* knock down decreases threshold to painful mechanical stimuli (Tulleuda et al., 2011; Castellanos et al., 2020).

TRESK is not the only K_2p_ channel involved as TASK3 (K_2__*p*_9) and TWIK1 (K_2p_1) are also down-regulated in DRG by SNI but K_2p_3 (TASK1) is not. Expression of K_2p_9 returns to normal within weeks, whereas K_2p_1 channels remain depleted for months (Pollema-Mays et al., 2013).

Although volatile anesthetics are known to activate K_2p_2, 3, 4, 9, and 18 (Gada and Plant, 2019) and this may relate to their clinical effectiveness, it is obviously impractical to use these drugs for long term control of chronic pain. There is, however, a recent report a novel TREK2/ K_2p_10.1 activator presently known as GI-530159. Although this substance decreases DRG excitability (Loucif et al., 2018), its possible effectiveness in pain models has not yet been reported.

## Physiological and Pathophysiological Regulation of K^+^ Channels in Primary Afferent Neurons

It is clear from the forgoing sections that K^+^ channel function is regulated by a variety of mechanisms, including trafficking, alternate splicing of gene products, protein–protein interactions, channel subunit interactions, as phosphorylation and interaction with membrane phospholipids. Channel expression can be altered by the action of transcription factors, microRNA and DNA methylation. All such processes are involved in alteration of K^+^ channel function in sensory neurons following injury (Uchida et al., 2010a,b; Kanda et al., 2011; Rose et al., 2011; Zhang X. L. et al., 2012; Sakai et al., 2013, 2017; Kuo et al., 2017; Zemel et al., 2017). These diverse cellular mechanisms are engaged by extracellular signals such as inflammatory mediators and growth factors released from the site of injury (Wagner and Myers, 1996a,b; Sorkin et al., 1997; Sommer et al., 1999; Cui et al., 2000; Scholz and Woolf, 2007; Takeda et al., 2008; Stemkowski and Smith, 2012a,b; Shinoda et al., 2019). Several lines of evidence collaborate the involvement of soluble mediators in injury-induced changes in DRG excitability and K^+^ channel function. It is for example, well established that peripheral nerve injury alters the properties of both severed and undamaged axons (Ma et al., 2003; Yang et al., 2018) and, as already mentioned (section “Spinal Cord Injury and Changes in Peripheral Neuron Excitability”), injury to the spinal cord *per se* can induce changes in DRG excitability and channel expression (Yang et al., 2014; Ritter et al., 2015a). Lastly the phenomenon of “mirror image pain” where unilateral injury provokes changes in contralateral nerves is well characterized. This phenomenon also involves the action of soluble inflammatory mediators and growth factors (Milligan et al., 2003; Cheng et al., 2014, 2015; Xie et al., 2016; Yuan et al., 2020).

### Cytokines in Neuropathic Pain

It is widely accepted that a transient injury-induced inflammatory event is the primary trigger to the onset of neuropathic pain (Watkins and Maier, 2002; Scholz and Woolf, 2007; Kawasaki et al., 2008a,b; Leung and Cahill, 2010; Gaudet et al., 2011; Stemkowski and Smith, 2012b; von Hehn et al., 2012; Noh et al., 2020). The crucial interplay between the nervous and immune systems (Ren and Torres, 2009) has led some authors to describe neuropathic pain as a neuroimmune disorder (Scholz and Woolf, 2007; Grace et al., 2014). It should also be noted that cytokine production is not wholly maladaptive as both interleukin-1β (IL-1β) and tumor necrosis factor-α (TNF-α) have been implicated in functional recovery after peripheral nerve injury (Nadeau et al., 2011). Although a multiplicity of inflammatory and anti-inflammatory cytokines, chemokines, eicosanoids, neuropeptides and growth factors have been implicated, my primary focus will be on the most extensively studied agents, i.e., IL-1β, TNF-α, NGF, and BDNF.

#### Interleukin 1β (IL-1β) and K^+^ Channels

Nerve injury orchestrates the release of IL-1β from mast cells, mononuclear cells, neutrophils, Schwann cells, fibroblasts, endothelial cells, satellite glial cells and neurons themselves (Sommer and Kress, 2004; Scholz and Woolf, 2007). A 10-fold increase in IL-1β protein expression in sciatic nerve is seen between 1 and 7 days after nerve injury (Nadeau et al., 2011) and impairment of interleukin-1 signaling following nerve injury by deletion of IL-1 receptor type 1 or transgenic over-expression of the physiological IL-1 receptor antagonist attenuates neuropathic pain, autotomy, and spontaneous ectopic neuronal activity (Wolf et al., 2006).

Acute or chronic exposure to IL-1β increases the excitability of DRG neurons (Binshtok et al., 2008; Stemkowski and Smith, 2012a; Stemkowski et al., 2015). The long-term actions of this particular cytokine are of special relevance as they are dominated by changes in K^+^ channel function. Thus, 5–6 days exposure of medium sized DRG neurons to 100pM IL-1*β* reduced the amplitudes of A-current, delayed rectifier and BK currents by 68, 64, and 36%, respectively. This likely reflected changes in K_v_1.2, 2.1, 2.2, 3.4, 4.2 and K_Ca_1.1. But only modest changes in HCN, Na^+^ and Ca^2+^ currents were seen. This action is neuron type-specific as small IB4-positive and large DRG neurons were little affected. Effects on small IB4-negative neurons were dominated by decrease in BK channel function (Noh et al., 2019). In our experiments, neurons were maintained in defined medium culture and exposed to IL-1β for 5–6 days to mimic the time course of cytokine increase seen after nerve injury (Stemkowski and Smith, 2012a; Stemkowski et al., 2015; Noh et al., 2019). One interesting finding that emerged from this series of experiments is that while effects of IL-1β on medium cell excitability returned to control levels following removal of cytokine for 3–4 days (Stemkowski et al., 2015), effects on small IB4-negative neurons were more persistent (Noh et al., 2019). This implies that IL-1β produces both acute effects on ion channel function as well as more enduring changes in neuronal phenotypic properties, perhaps mediated at the transcriptional level. The idea that decreased K^+^ channel function in nerve terminals can affect neurotransmitter release from primary afferent terminals has already been alluded to Furukawa et al. (2008) and Muqeem et al. (2018). This may relate to the observation that 6–8 days exposure to IL-1β increases sEPSC amplitude in *substantia gelatinosa* neurons (Gustafson-Vickers et al., 2008), perhaps reflecting an action of the cytokine on K^+^ channels in primary afferent terminals.

#### Tumor Necrosis Factor-α (TNF-α) and K^+^ Channels

The evidence implicating TNF-α in neuropathic pain parallels that described above for IL-1β (Leung and Cahill, 2010). Thus it is detected at the site of injury following CCI (George et al., 1999), intra-sciatic injection produces pain (Wagner and Myers, 1996a) and injury induced hyperalgesia is abrogated following inhibition of TNF-α synthesis with thalidomide (George et al., 2000). The TNF-α antagonist etanercept, similarly blocks tactile allodynia in diabetic mice (Dogrul et al., 2011). Unlike IL-1β, most actions of TNF-α involve modifications of Na^+^ channel function (Jin and Gereau, 2006) rather than any effect on K^+^ channels (He et al., 2010).

### Growth Factors in Neuropathic Pain

Nerve injury increases levels of BDNF and NGF in peripheral nerves and attenuation of their actions leads to attenuation of allodynia and other signs of neuropathic pain (Herzberg et al., 1997; Theodosiou et al., 1999; Zhou et al., 2000; Pezet and McMahon, 2006). Neurotrophins alter expression, posttranslational modification and trafficking of TRPV1 and voltage gated sodium channels (Dib-Hajj et al., 1998, 2010; Mantyh et al., 2011).

#### NGF and K^+^ Channels

Ligation or crush of the sciatic nerve reduces voltage-gated, transient and persistent K^+^ currents in large cutaneous afferent DRG neurons but these are restored by *in vivo* administration of NGF (Everill and Kocsis, 2000). NGF has also been reported to increase immunoreactivity for small but not intermediate Ca^2+^ gated K^+^ channels in injured human sensory neurons (Boettger et al., 2002). Other work has, however, suggested that NGF working through the p75 neurotrophin receptor reduces K^+^ current in small-diameter capsaicin-sensitive sensory neurons (Zhang Y. H. et al., 2012). Although NGF inhibits K_v_7 channels in sympathetic neurons (Jia et al., 2008), it remains to be determined whether these channels are similarly affected in DRG neurons. These divergent effects of NGF on K^+^ channels may parallel its actions on Na^+^ channels, which involve increased activity of some channel types in some neuron types and decreased activity in others (Black et al., 1997; Dib-Hajj et al., 1998; Fjell et al., 1999). Despite this, antagonism of the actions of NGF is generally effective in alleviating pain (Ro et al., 1999; Djouhri, 2016) and a loss of function mutation in TrkA leads to congenital insensitivity to pain (Indo, 2002). It is likely therefore that NGF’s actions in generating allodynia involve a decrease K^+^ currents and an increase certain voltage-gated Na^+^ currents.

#### BDNF and K^+^ Channels

The role of microglia derived BDNF in central sensitization is well established (Coull et al., 2005; Lu et al., 2007, 2009; Smith, 2014; Boakye et al., 2019). Since injury-induced release of NGF increases expression of BDNF in nociceptors (Pezet and McMahon, 2006), it has also been implicated in peripheral pain mechanisms. BDNF levels are increased in DRG following nerve injury and this may lead to a decrease in BK currents (Cao et al., 2012). It also reduces expression of K_v_4.2 and K_v_4.3 genes in whole DRG (Park et al., 2003) whereas BDNF antibodies or a TrkB blocker increases Kv4.2 and Kv4.3 mRNA and A-type K^+^current (Cao et al., 2010). Interestingly BDNF also increases spontaneous excitatory synaptic activity in the spinal dorsal horn (Lu et al., 2007, 2009) it is therefore tempting to speculate that this may reflect downregulation of K_v_4.2/4.3 in primary afferent terminals.

### Other Soluble Factors Controlling K^+^ Channel Function

Several other soluble mediators have been implicated in signaling between the inflammatory response and the initiation of neuropathic pain. These include interferon γ (Vikman et al., 2003), interleukin 6 (IL-6) (Xu et al., 1997; Arruda et al., 1998), glial cell line derived neurotrophic factor (GDNF) (Cummins et al., 2000; Leffler et al., 2002), fibroblast growth factor (bFGF), transforming growth factor-beta1 (TGF-beta) (DeLeo et al., 1997; Zhu et al., 2012a) and prostaglandins (Syriatowicz et al., 1999; Kanda et al., 2017). Despite the identification of a considerable number of extracellular mediators, it is interesting to note the similarity in the transduction processes they activate; rasMAP kinase and p38 kinases are frequently involved. Since K^+^ channels function is clearly affected by IL-1β and BDNF, it is likely that interferon γ, IL-6, GDNF, bFGF, TGF-beta and prostaglandins work through similar transduction processes to thereby regulate K^+^ channel function in the context of neuropathic pain. Available evidence to support this contention comes from the observation that GDNF increases spike width of small IB4-negative DRG neurons (Stemkowski et al., 2015) and prostaglandin E2 attenuates the slow action potential afterhyperpolarization in DRG neurons in culture (Gold et al., 1996b).

## K^+^ Channelopathy and Neuropathic Pain

The relationship between K^+^ channel dysfunction and neuropathic pain in animal models is borne out in the clinic where certain neurological disorders that involve peripheral hyperexcitability and pain also involve altered K^+^ channel function. For example, K_v_ antibodies have been detected in acquired neuromyotonia patients (Hart et al., 2002) and treatment of murine DRG neurons with these antibodies increased their excitability (Shillito et al., 1995). Other patients with acquired neuromyotonia or Morvan’s syndrome express antibodies to K_v_1 channel complex proteins leucine-rich, glioma inactivated 1 protein and contactin-associated protein-2 (Irani et al., 2010).

Gain-of-function mutations of the Na_v_1.7 sodium channel produce a painful inherited condition known as erythromelalgia (Dib-Hajj et al., 2005). Although individuals afflicted with this chronic “burning man syndrome” suffer debilitating chronic pain, a subset of individuals who have this mutation in Na_v_1.7 are relatively pain resistant. This has been ascribed to an additional gain of function mutation in *KCNQ* which encodes K_v_7.2 (Mis et al., 2019). This study illustrates how differences in cation channel expression in sensory neurons can contribute to inter-individual differences in pain (Waxman, 2019).

It should be mentioned that K^+^ channels mutations are very rare in the human population and are often manifest as epilepsy or ataxia (Kullmann, 2010). There is, however, an emerging literature on K^+^ channelopathies and migraine (Cregg et al., 2010; Lafreniere and Rouleau, 2012; Albury et al., 2017), perhaps improved understanding of peripheral neuropathy will contribute to better understanding of migraine and *vice versa.*

## Conclusion

Despite issues associated with *bona fide* pain measurement (section “How Is Pain Measured?”), the use of different injury models (see section “Lost in Translation”), differences in neuron classifications used by different groups, and influences of genetics and epigenetics, there can be little doubt that dysfunction of K^+^ channels in primary afferents is involved in both the onset and persistence of neuropathic pain. It is also clear that perturbation of K^+^ channel function contributes to the development and persistence of neuropathic pain as a result of increased excitability of primary afferent neurons. This leads to spontaneous activity, increased axonal conduction and increased neurotransmitter release from primary afferent terminals (Furukawa et al., 2008; Barkai et al., 2017; Evely et al., 2017; Muqeem et al., 2018; Zhang et al., 2018).

It has long been recognized that different types of DRG neuron express different palates of K^+^ channels (Gold et al., 1996a) and in general, nerve injury promotes decreases in their functionality. Different changes are seen in different DRG neuron types; some channels are affected whereas others are not. Table 1 summarizes how various types of nerve injury affect various channel types and Table 2 summarizes changes seen in different DRG neuron types in response to injury or the actions of inflammatory mediators.

The observed effects of nerve injury involve changes involve multiple processes affecting K^+^ channel expression, epigenetic modulation and trafficking. This is illustrated by the multiplicity of processes regulating K_v_ 1.2 delayed rectifier channels (see section “Effects of Nerve Injury on Delayed Rectifier K^+^ Currents and Channels in DRG Neurons”). These processes are initiated by the release of inflammatory mediators and growth factors following Wallarian degeneration at the site of injury.

One interesting point that emerges from the literature reviewed is that selective perturbation of more or less any single DRG K^+^ channel type can lead to chronic pain. As well as underlining the role K^+^ channels in neuropathic pain, this observation implies that function of all K^+^ channels has to be unperturbed to prevent pain onset. In other words “everything has to be right with K^+^ channels or else pain will occur.”

The findings of cellular and *ex vivo* studies need to be correlated with behavioral studies in order to verify K^+^ channel dysfunction with signs of pain. *In vivo* studies in which channel function is augmented or impaired implicate delayed rectifiers, A-currents, M-currents, BK currents, K_ATP_ channels and leak channels in the etiology of neuropathic pain. It should be noted, however, that most of the *in vivo* studies involve global perturbation of channels throughout the nervous system, including those in glial cells and in central neurons. It is uncertain therefore whether behavioral responses are only attributable to changes in K^+^ channels in peripheral neurons. These issues will be resolved as improved methods for selectively altering channel expression within different DRG neuron types are applied. This has already been done with selective optogenetic attenuation of function of Na_v_1.8 expressing nociceptors (Daou et al., 2016).

Despite these difficulties, recognition of the importance of K^+^ channel dysfunction has led to considerable interest in the potential role of K^+^ channel activators in pain management (Ocana et al., 2004; Busserolles et al., 2016; Barrese et al., 2018; Zemel et al., 2018; Abd-Elsayed et al., 2019; Gada and Plant, 2019). Unfortunately, this promise is yet to be fulfilled either because prospective agents do not produce statistically significant effects in sufficiently large populations (Yekkirala et al., 2017) or because unexpected toxicities limit long term drug use. It should be noted, however, that some of the actions of gabapentinoids and cannabinoids that display efficacy in neuropathic pain may be mediated by augmentation of K^+^ channel function (Manville and Abbott, 2018; Li Y. et al., 2019). Given the improved understanding of cellular processes controlling channel function, it may be useful to target upstream mediators such as the histone methylase G9a which controls the function of several K^+^ channel types (Laumet et al., 2015).

On a more positive note, however, K^+^ channel activators may play a role with the advent of personalized medicine approaches to pain management (Bannister et al., 2020). Determination of the sensory phenotypes and genetics of individual patients may dictate drug effectiveness and define therapeutic approaches. K^+^ channel activators should not be overlooked in this regard.

## Author Contributions

The author confirms being the sole contributor of this work and has approved it for publication.

## Conflict of Interest

The author declares that the research was conducted in the absence of any commercial or financial relationships that could be construed as a potential conflict of interest.
